# Distinct patterns of proteostasis network gene expression are associated with different prognoses in melanoma patients

**DOI:** 10.1038/s41598-023-50640-0

**Published:** 2024-01-02

**Authors:** Rachel Wellman, Daniel Jacobson, Maria Secrier, John Labbadia

**Affiliations:** 1 Division of Biosciences, Department of Genetics, Evolution and Environment, Institute of Healthy Ageing, University College London, London, UK; 2 Division of Biosciences, Department of Genetics, Evolution and Environment, UCL Genetics Institute, University College London, London, UK; 3 UCL Cancer Institute, University College London, London, UK

**Keywords:** Systems biology, Regulatory networks

## Abstract

The proteostasis network (PN) is a collection of protein folding and degradation pathways that spans cellular compartments and acts to preserve the integrity of the proteome. The differential expression of PN genes is a hallmark of many cancers, and the inhibition of protein quality control factors is an effective way to slow cancer cell growth. However, little is known about how the expression of PN genes differs between patients and how this impacts survival outcomes. To address this, we applied unbiased hierarchical clustering to gene expression data obtained from primary and metastatic cutaneous melanoma (CM) samples and found that two distinct groups of individuals emerge across each sample type. These patient groups are distinguished by the differential expression of genes encoding ATP-dependent and ATP-independent chaperones, and proteasomal subunits. Differences in PN gene expression were associated with increased levels of the transcription factors, *MEF2A, SP4, ZFX, CREB1 and ATF2,* as well as markedly different survival outcomes. However, surprisingly, similar PN alterations in primary and metastatic samples were associated with discordant survival outcomes in patients. Our findings reveal that the expression of PN genes demarcates CM patients and highlights several new proteostasis sub-networks that could be targeted for more effective suppression of CM within specific individuals.

## Introduction

Cutaneous melanoma (CM), the deadliest form of skin cancer, occurs following the malignant transformation of melanocytes^1^. Exposure to UV radiation is the most significant risk factor, with 75% of cases being attributed to UV exposure^2^. The disease is becoming increasingly common as numbers of people who have low skin pigmentation travel to and live in countries with high levels of sunlight, and as use of UV sun beds continues. The International Agency for Research on Cancer (part of the World Health Organisation) has predicted that between 2020 and 2040 numbers of new cases of melanoma will increase by 50% and deaths will increase by 68%^3^. This not only causes human suffering but also places an increasing financial burden on public health services.

New treatments for melanoma, particularly immunotherapy and targeted therapies, have improved prognosis, and many cases are now treated successfully, with mortality rates in the United States declining by around 4% per year since 2015, despite incidence increasing^4^. However, heterogeneity within and between tumours, and a tendency towards increased drug resistance, lead to relatively high rates of recurrence and fatality. This variability in clinical outcomes has motivated extensive research to identify clinical and genetic factors that may aid prognosis and the development of new treatments, as well as the improved targeting of existing treatments.

A key feature of many cancers, including melanoma, is a dependency on the protein homeostasis (proteostasis) network (PN) for tumorigenesis and growth. The PN is a large and intricate network of protein quality control pathways and stress responses that maintains the quality, quantity and location of a cell’s proteins in the face of damage caused by cytotoxic stressors, including toxic chemicals, radiation and ageing^5^. Components of the PN work together to maintain proteostasis by folding and localising new proteins, unfolding and refolding damaged proteins and degrading any proteins that are beyond repair^5^, thereby protecting the integrity of the proteome and ensuring cell viability.

In CM, as in other cancers, the PN is extensively remodelled to prevent proteotoxicity that would otherwise be caused by elevated protein load, stoichiometric imbalances of protein complexes and increased incidence of mutant proteins that arise as a result of UV-induced molecular damage^1^. In particular, CM cells require chaperones, such as HSP90 and HSP70, the expression of which increases in CM compared to normal melanocytes^6^, activation of the Endoplasmic Reticulum (ER) Unfolded Protein Response (UPR^ER^)^7^, and augmented ubiquitin/SUMO proteasome systems^8,9^.

The increased reliance of cancer cells on the maintenance of proteostasis for survival has led to the development of drugs that inhibit individual components of the PN. These may be used alone or in combination with drugs which increase proteotoxicity. For example, Bortezomib inhibits the proteasome and is currently used in the treatment of myeloma^10^ and mantle cell lymphoma^11,12^. Furthermore, in vitro research has been carried out into the potential to use Bortezomib in combination with other drugs in the treatment of CM^10,13^. Several inhibitors of HSP90 that are effective in CM cell lines have also been identified^14^ and research has also been conducted on the use of other PN inhibitors as sole or adjuvant drugs in the treatment of CM. Experiments using melanoma cell lines have identified HSP70 and HSP110 inhibitors that may be repurposed to treat CM^15^ and a small clinical trial of combined BRAF and HSP90 inhibition in patients with unresectable BRAF V600E mutant melanoma had promising results^16^. However, at present no PN inhibitory drugs are approved for the treatment of CM. Elements of the PN have also been identified as prognostic markers in CM, for example the expression of proteasome activator subunits PSME1, 2 and 3^17^.

While several studies have considered the role of individual elements of the PN on CM incidence and progression, less is known about the wider pattern of PN gene expression within primary and metastatic tumours and whether the heterogeneity of PN gene expression across patients may influence survival outcomes. To address this, we investigated the expression of 428 “core PN” genes within primary and metastatic samples from CM cohorts obtained from the Cancer Genome Atlas (TCGA). By clustering samples based on PN gene expression, we observed that distinct PN gene expression profiles correlate with patient survival outcomes and provide further insights into the cellular programmes modulating these phenotypes.

## Results

### Primary and metastatic melanoma samples exhibit two distinct patterns of proteostasis network gene expression

Given that the maintenance of proteostasis is crucial for the survival of CM cells, we hypothesised that the transcriptional remodelling of the proteostasis network (PN) may be associated with clinical outcomes in CM patients. To test this, we compiled a list of 428 core PN genes and compared expression changes across a panel of 103 CM primary and 356 metastatic samples, using existing RNA-sequencing data available from The Cancer Genome Atlas (TCGA). The PN genes selected encompassed genes with roles in folding of native proteins and refolding of non-native proteins (molecular chaperones and co-chaperones), ubiquitination and de-ubiquitination (E1, E2, E3, DUBs), proteasomal degradation (19S and 20S) and autophagy (Supplementary Table 1).

**Figure 1:**
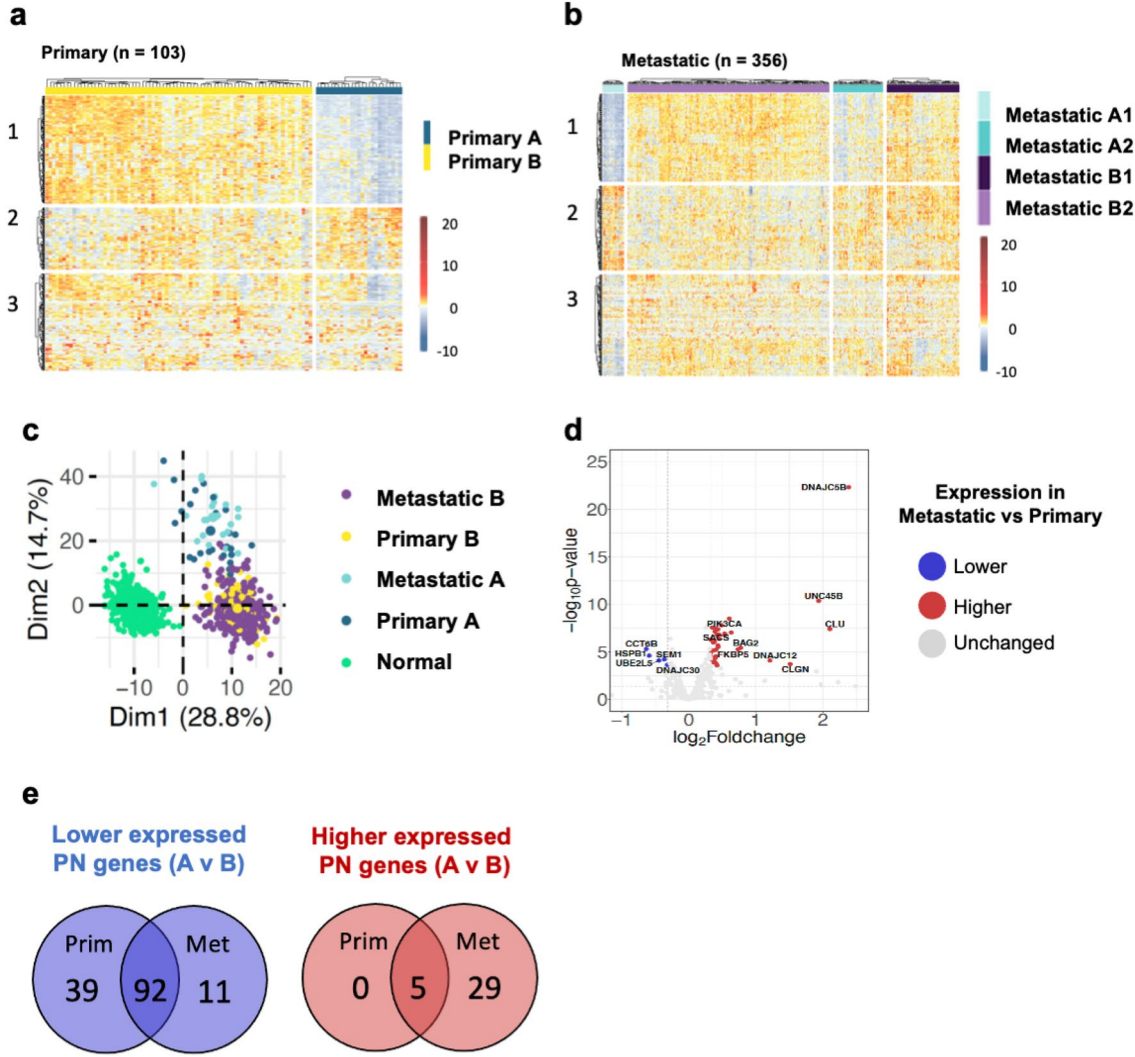
Two distinct patterns of PN gene expression are observed across primary and metastatic CM samples. (**a**, **b**) Proteostasis network gene expression (corrected for tumour purity) in (**a**) primary and (**b**) metastatic cutaneous melanoma (CM) samples clustered using Ward’s hierarchical agglomerative clustering method. (**c**) Principal component analysis of PN gene expression in normal, primary and metastatic samples. (**d**) Volcano plot of differences in PN gene expression between TCGA primary and metastatic samples. (**e**) Venn diagram showing numbers of PN genes that have lower or higher expression in sample group A than in sample group B in primary and metastatic CM samples.

Clustering of samples based on similarity of gene expression demonstrated that the cohorts of primary and metastatic CM samples can be divided into clearly differentiated groups (Supplementary Fig. 1a–b). To determine whether this reflected differences in cellular composition between samples, we compared levels of immune cell infiltration using Consensus-TME^18^. We found that the levels of many types of immune cells differed between the groups (Supplementary Fig. 1c–d & Supplementary Table 2). Therefore, we adjusted our gene expression data to correct for levels of tumour purity^19^ and re-clustered the samples based on the adjusted expression values. Following this adjustment, two primary samples moved from one cluster to the other (Supplementary Fig. 1e). Both the primary and metastatic samples still clustered into distinct groups, which we called ‘Primary A’ (n = 25) and ‘Primary B’ (n = 78) (Fig. 1a) and ‘Metastatic A1’ (n = 22), ‘Metastatic A2’ (n = 51), ‘Metastatic B1’ (n = 75) and ‘Metastatic B2’ (n = 208) (Fig. 1b). In order to more confidently investigate the causes and effects of altered PN gene expression in CM patients, we combined the two sample groups with lower expression of the genes in cluster 1, Metastatic A1 and Metastatic A2, into a larger group termed ‘Metastatic A’, and combined the two groups with higher expression of these genes, Metastatic B1 and Metastatic B2, into a single group termed ‘Metastatic B’ (Supplementary Fig. 1f).

An analysis of demographic data related to the patients in each group showed that differences in gender, ethnicity, age or stage at initial diagnosis were unlikely to explain the differences in PN gene expression between the groups, although it should be noted that there was a small increase (*p* = 0.043) in the proportion of female patients present in Metastatic B (Supplementary Fig. 2a–d). Similarly, all primary and metastatic groups exhibited mutational signatures commonly associated with UV damage and skin cancer^20^ but did not show discernible differences in the proportion of contribution of other mutational signatures (Supplementary Fig. 2e).

To determine whether there were similarities in expression patterns between Primary A and Metastatic A, and between Primary B and Metastatic B, and to compare their gene expression with that of normal skin cells, we carried out principal component analysis on normalised TCGA (cancer) and GTEX (normal) data. Both Primary and Metastatic A, and Primary and Metastatic B, showed high similarity to one another (Fig. 1c). Furthermore, all 4 groups were markedly different from normal cells (Fig. 1c), demonstrating that the expression patterns of the different CM groups have diverged markedly from those of non-cancerous cells. The majority of PN genes were expressed at similar levels in primary and metastasised tissues; however, several PN genes did show a marked difference in expression, including several DNAJ chaperones (*DNAJC5b, DNAJC12, DNAJC30, SACS*), the small heat shock protein *HSPB1*, the TRiC subunit *CCT6B* and components of the ubiquitin proteasome system (*UBE2L5, SEM1*) (Fig. 1d).

Of the 428 PN genes investigated, we found that 136 were differentially expressed in the primary tumours, thus driving the differences between groups A and B. Similarly, 137 PN genes were differentially expressed in the metastases. Among these, 92 genes were expressed at lower levels in both primary and metastatic group A compared to group B (Fig. 1e and Supplementary Table 3). Similarly, 5 genes exhibited higher expression in both primary and metastatic (34 genes total) group A versus group B (Fig. 1e and Supplementary Table 3). The primary group had 131 PN genes that had lower expression in group A, and 5 that had higher expression in group A. The metastatic group had 103 PN genes that had lower expression in group A, and 34 that had higher expression in group A. No PN gene exhibited discordant expression differences between primary and metastatic sample groups A and B.

Together these observations show primary and metastatic skin cancer samples exhibit significantly different patterns of PN gene expression across patients, and that similar sets of PN genes are differentially expressed between corresponding primary and metastatic CM groups.

### The expression of PN genes is disproportionately altered between CM patients compared to the total transcriptome and is not reflected in all cancers

**Figure 2:**
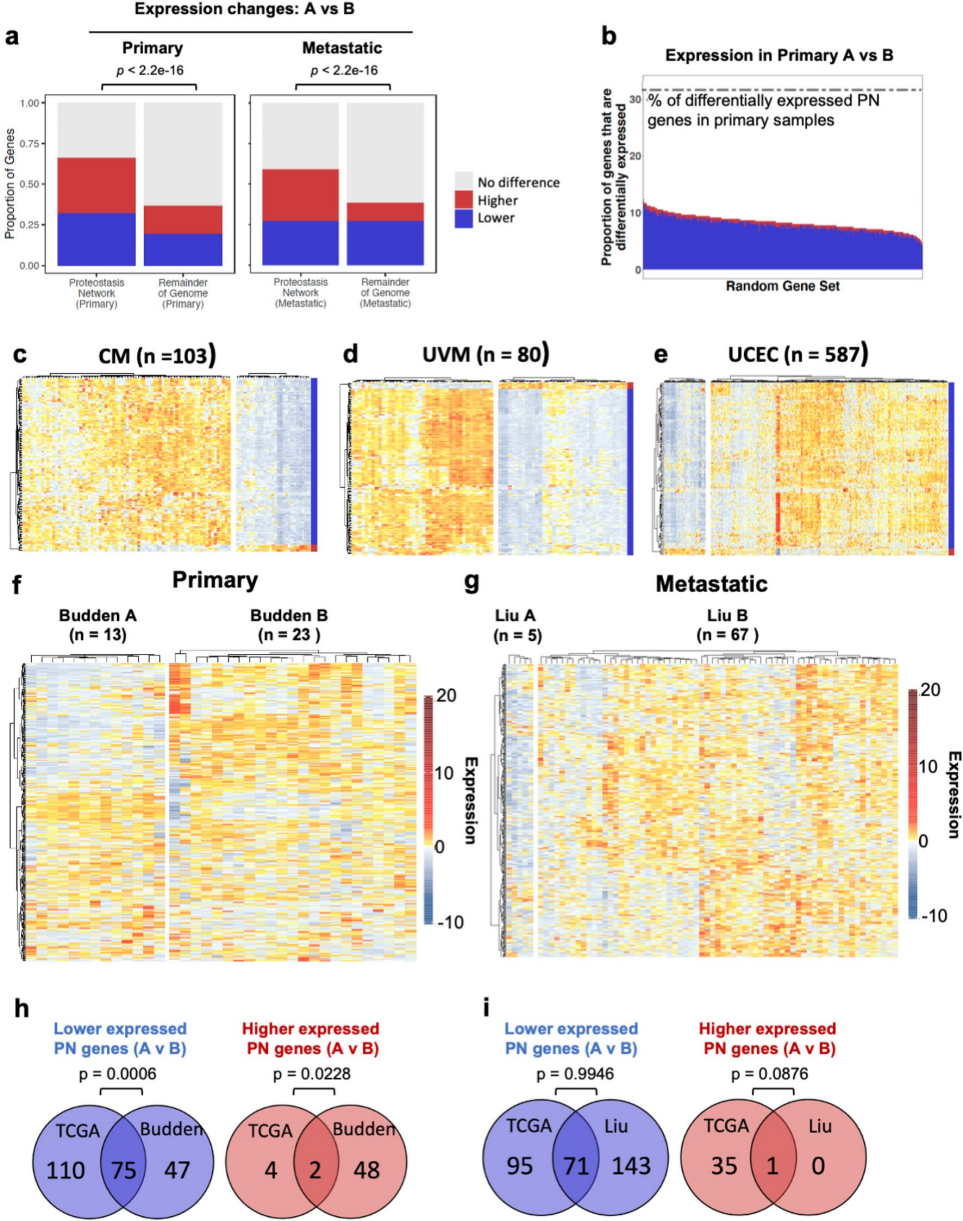
The proportion of PN genes exhibiting differential expression across CM samples is greater than that observed across the rest of the genome. (**a**) Proportion of Proteostasis Network (PN) genes or non-PN genes showing significantly different expression between cutaneous melanoma (CM) primary and metastatic groups A and B (*p*-value < 0.05 calculated by Student’s T-test and DEseq2, adjusted *p*-value < 0.1 calculated by Benjamini Hochberg correction). (**b**) Proportion of random gene sets that exhibit differential expression between primary groups A and B (*p*-value < 0.05 calculated by Student’s T-test and DEseq2, adjusted *p*-value < 0.1 calculated by Benjamini Hochberg correction). (**c**, **d**) Expression of PN genes clustered using Ward’s hierarchical agglomerative clustering method in (**c**) primary cutaneous melanoma (CM), (**d**) uveal melanoma (UVM) and (**e**) uterine corpus endometrial carcinoma (UCEC). (**f**, **g**) Expression of PN genes in (**f**) primary (Budden) and (**g**) metastatic (Liu) validation cohorts clustered using Ward’s hierarchical agglomerative clustering method. (**h**, **i**) Numbers of genes exhibiting higher or lower expression in group A than group B in (**h**) primary TCGA and Budden samples and (**i**) metastatic TCGA and Liu samples. P-values were calculated as the probability of achieving the same (or greater) degree of overlap in 5000 simulations of two randomly selected lists of PN genes of equal sizes to those being compared between our TCGA/Budden and TCGA/Liu cohorts.

To determine whether the expression of PN genes was preferentially altered compared to non-PN genes, we compared the expression of all PN and non-PN genes across our Primary and Metastatic CM groups. In both sample types, the proportion of PN genes that were differentially expressed between groups A and B was approximately twofold higher than the proportion of non-PN genes that were differentially expressed based on *p* values < 0.05 and adjusted *p* values < 0.1 calculated by both Student’s T test and DESeq2 (Fig. 2a). In addition, we analysed 1000 sets of 428 randomly selected genes to determine the proportion exhibiting significantly different expression between our groups (*p* values < 0.05 and adjusted *p* values < 0.1 calculated by both Student’s T test and DESeq2). We found that 6.53% of randomly selected genes were differentially regulated in primary and metastatic samples, as compared to 31.8% and 32.0% of PN genes, respectively (Fig. 2b, and Supplementary Fig. 3a). These observations confirm that in both primary and metastatic tissues, the expression of PN genes is disproportionately altered across patients.

We next wanted to determine whether the pattern of PN gene expression observed across individuals is specific to CM, and whether similar heterogeneity in the expression of PN genes is also seen in other patient cohorts/data sets. First, the expression of PN genes was assessed in each of the other 32 cancers represented in TCGA. Samples in each study were divided into two groups based on their PN gene expression profiles using Ward’s hierarchical agglomerative clustering method and the percentage of genes with significantly different expression between the two groups was counted. Both uveal melanoma (UVM) (n = 80) and uterine corpus endometrial carcinoma (UCEC) (n = 587) displayed almost identical changes in PN gene expression across patients to those observed in CM samples (Fig. 2c–e, Supplementary Fig. 3b). In contrast, only 25% of PN genes that showed differences in expression across CM patients, also did so in uterine carcinosarcoma (UCS) (n = 56) and cholangiocarcinoma (CHOL) (n = 46). All other cancers tested, including Kidney renal clear cell carcinoma (KIRC) (n = 611) and Prostate adenocarcinoma (PRAD) (n = 551) exhibited between 75 and 30% similarity with the PN gene expression changes observed in CM samples (Supplementary Fig. 3c–f and Supplementary Table 4). Our findings show that differential transcriptional remodelling of the PN is observed in cancers beyond CM, but that the patterns of PN gene expression changes are most similar in uveal and cutaneous melanomas.

Finally, we wanted to address whether the PN gene expression changes we observed in CM were limited to samples within TCGA or were a more general phenomenon across patients. In order to validate our findings, we obtained gene expression data from two published studies, one of primary cases by Budden et al. (n = 34) ^21^ and one metastatic by Liu et al. (n = 72)^22^. In each case, the pattern of PN gene expression was similar, though not identical to that observed in the TCGA samples, with distinct sub-groups distinguished by similar features (Fig. 2f and g). We also compared how many of the genes that were differentially expressed in the TCGA cohort, as analysed using Student’s T-test, were differentially expressed in the Budden and Liu cohorts. 75 of the genes that had lower expression in TCGA Primary A also had lower expression in Budden A, and 2 that had higher expression in TCGA Primary A also had higher expression in Budden A (Fig. 2h). Similarly, 71 of the genes that had lower expression in TCGA Metastatic A also had lower expression in Liu A, and 1 that had higher expression in TCGA Metastatic A was also more highly expressed in Liu A (Fig. 2i). Comparing the overlaps of randomly selected PN genes with those observed between our TCGA/Budden and TCGA/Liu cohorts revealed that the overlaps observed for lower (*p* = 0.0006) and higher (*p* = 0.0228) expressed PN genes in primary samples are unlikely to occur by chance (Fig. 2h). In contrast, we found that this was not the case for our TCGA/Liu overlaps (Lower, *p* = 0.9946; Higher, *p* = 0.0876) (Fig. 2i). These data suggest that the demarcation of CM patients by PN gene expression is a general phenomenon in primary tissue samples. In contrast, while sub-groups of individuals can be distinguished by the expression of PN genes in two independent metastatic CM cohorts, the specific PN genes involved may differ.

### CM sub-groups are distinguished by a transcriptional shift from ATP-dependent to non-ATP dependent proteostasis systems

**Figure 3:**
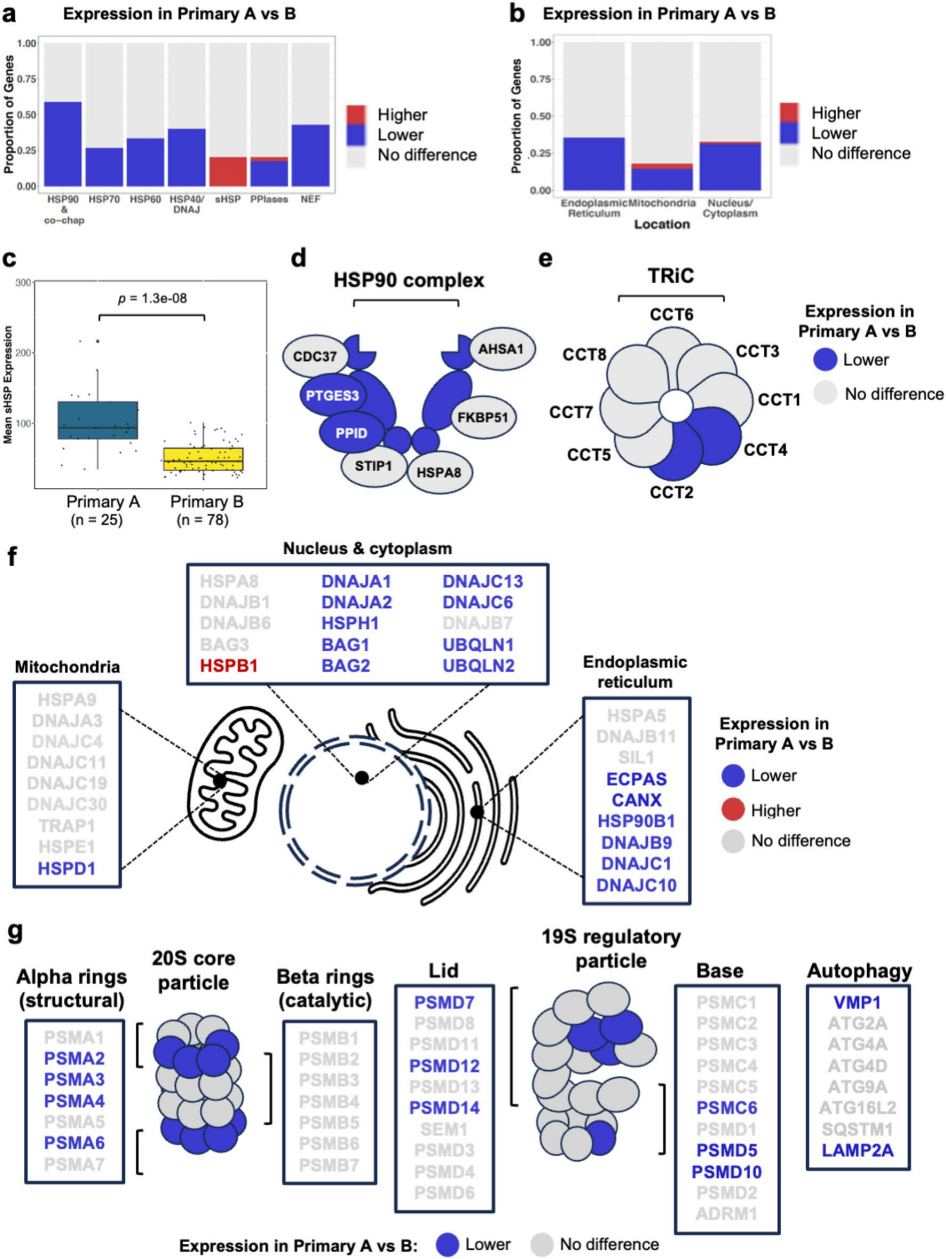
Specific components of the sHSP, HSP90, HSP60, HSP70/DNAJ and proteasome systems are differentially expressed across primary CM samples. (**a**) Proportion of genes within each Proteostasis Network (PN) sub-group showing significantly altered expression between primary groups A and B (*p*-value < 0.05 calculated by Student’s T-test and DEseq2, adjusted *p*-value < 0.1 calculated by Benjamini Hochberg correction). (**b**) Proportion of PN genes within each sub-cellular compartment showing significantly altered expression between primary groups A and B (*p*-value < 0.05 calculated by Student’s T-test and DEseq2, adjusted *p*-value < 0.1 calculated by Benjamini Hochberg correction). (**c**) Mean expression of sHSP genes in primary sample groups. *P*-values were calculated using Student’s t-test. Boxes indicate the interquartile range (IQR), the upper whisker extends to the largest value that is less than (third quartile + (1.5 * IQR)). The lower whisker extends to the smallest value that is greater than (first quartile − (1.5 * IQR)). (**d**–**g**) Cartoons highlighting the PN components that exhibit differential expression between Primary A and B among (**d**) HSP90 and co-chaperones, (**e**) CCT/TRIC subunits (**f**) core chaperones and co-chaperones of sub-cellular compartments and (**g**) proteasome core and regulatory particle subunits and autophagy components.

To understand how the differential pattern of PN gene expression across our samples might affect proteome management strategies, we calculated the proportion of PN genes within each chaperone family (HSP70, HSP90, HSP40, HSP60, PPIases, NEFs and sHSPs) showing lower, higher, or unchanged expression between our primary and metastatic sample groups. We found that all chaperone families examined contained differentially expressed PN genes in both primary and metastatic samples (Fig. 3a and Supplementary Fig. 4a). Similarly, PN gene expression was affected across subcellular locations, with comparable proportions of up and down regulated PN genes associated with the nucleus/cytoplasm, mitochondria or endoplasmic reticulum (ER) in primary or metastatic samples (Fig. 3b and Supplementary Fig. 4b).

Strikingly, genes encoding small heat shock proteins (sHSPs*/HSPB1*) exhibited higher expression in group A compared to group B in both primary and metastatic samples (Fig. 3a and Supplementary Fig. 4a), with the mean expression of all sHSPs also higher in Primary and Metastatic group A than B (Fig. 3c and Supplementary Fig. 4c). By contrast, the expression of core components of ATP-dependent chaperone machines in the cytosol/nucleus (*HSP90AA1/HSP90, CCT2, CCT4*), mitochondria (*HSPD1/HSP60*) and endoplasmic reticulum (*HSP90B1*) tended to have lower expression in primary/metastatic group A compared to group B (Fig. 3d and e and Supplementary Fig. 4d and e). Lower expression was also observed in genes encoding central components of the disaggregase machinery (*HSPH/HSP110*, *DNAJA1* and *DNAJA2*) in primary, but not metastatic, group A. However, major cytosolic (*HSPA8/HSC70*), mitochondrial (*HSPA9/mtHSP70*) and ER (*HSPA5/BiP*) HSP70s were all expressed at the same levels between sample groups ((Fisher’s exact test *p* > 0.05, Fig. 3f and Supplementary Fig. 4f).

In addition to differential expression of molecular chaperones and co-chaperones, we also observed lower expression of genes encoding subunits of the alpha-ring of the 20S proteasome core (*PSMA2, PSMA3, PSMA4 and PSMA6*) and components of the base and lid of the 19S regulatory particle (*PSMC6, PSMD7, PSMD12* and *PSMD14*) in primary group A (Fig. 3g). Similar changes were also observed in metastatic group A; however, in contrast to CM samples from primary tumours, some components of the base (*PSMD2* and *ADRM1*) and lid (*PSMD8*) were expressed at higher levels in metastatic group A, possibly as part of a compensatory mechanism for the lower expression of the other PN components identified here (Supplementary Fig. 4d–g).

Together our data suggest that CM cells/tissues employ one of two different strategies to maintain proteostasis: either a canonical ATP-dependent approach, utilising HSP70/HSP90/HSP60 folding machines and 26S proteasome activity, or a primarily non-ATP dependent approach, reliant on elevated levels of small heat shock proteins. Furthermore, our results suggest that while both primary and metastasised tumours can adopt different strategies for the maintenance of proteostasis, key differences between the tumour types are observed. These include differences in HSP90 co-chaperones, TRiC subunits, disaggregase subunits, ER chaperones, subunits of the proteasome and components of autophagy (Fig. 3d–g and Supplementary Fig. 4d–g).

### Alternative strategies for the maintenance of proteostasis are associated with different survival outcomes and clinical attributes in CM

To determine whether the differential remodelling of the PN across skin cancer cells is associated with differences in prognosis, we compared disease specific survival (DSS) outcomes of patients in Primary/Metastatic A with those of individuals in Primary/Metastatic B. We also compared disease specific survival in patients in the subgroups, Metastatic A1, A2, B1 and B2. For primary sample patients, survival over three years following diagnosis was analysed, as long-term survival data was not available (with the mean period between diagnosis and last contact or death being 1.4 years and the maximum being 5 years (Supplementary Fig. 5a)). For metastatic sample patients, survival over a 31-year period was monitored as longer-term survival data was available (with mean period from diagnosis to last contact or death being 6.4 years and the maximum being 31 years (Supplementary Fig. 5a).

**Figure 4:**
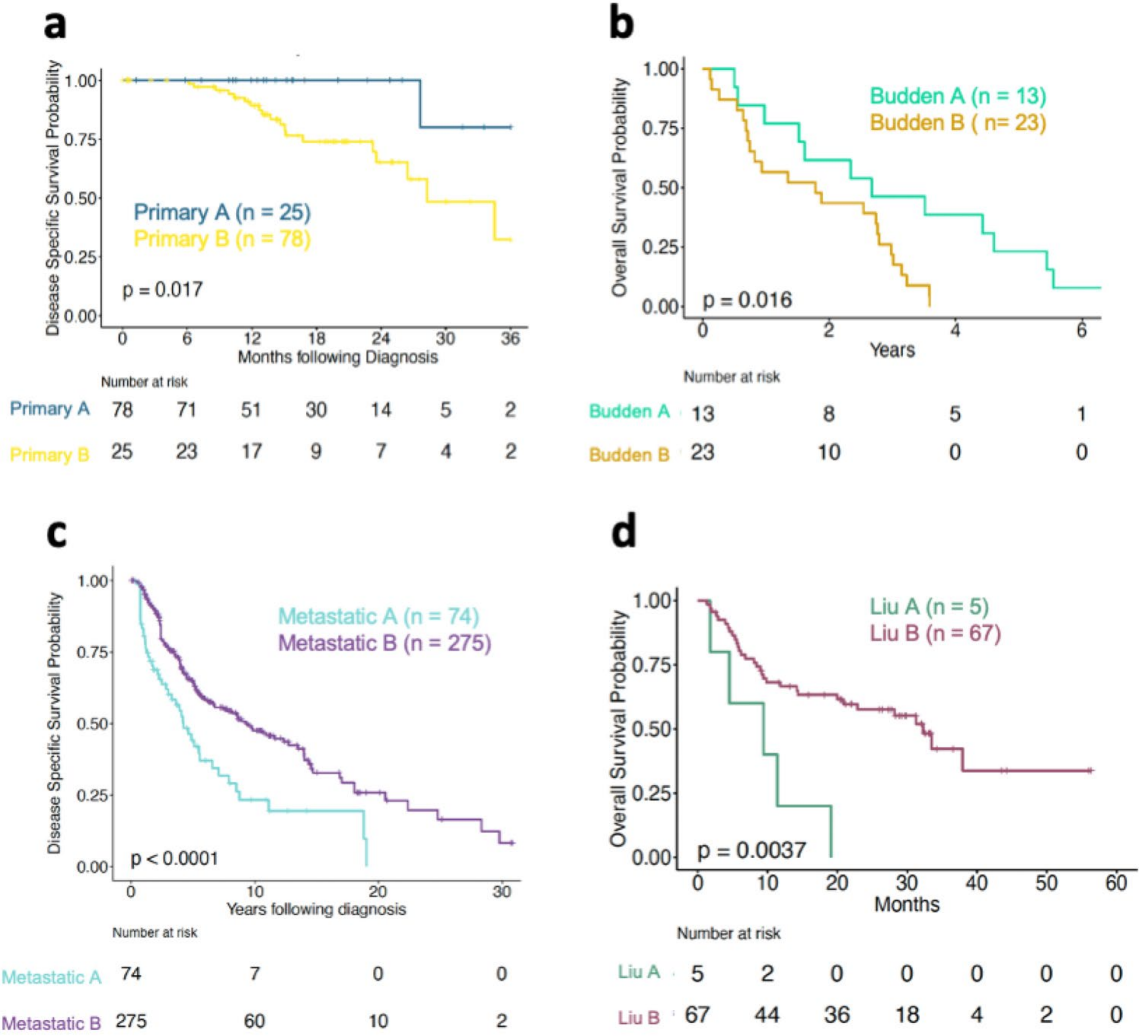
The differential expression of PN genes across primary and metastatic CM samples is associated with altered survival outcomes in patients. (**a**) Disease-specific survival curves and hazard tables for cutaneous melanoma (CM) patients in primary groups A and B of the TCGA cohort (3 years following diagnosis). (**b**) Overall survival curves and hazard tables for patients in Budden validation cohort (6 years following diagnosis). (**c**) Disease-specific survival curves and hazard tables for patients in metastatic groups A and B of the TCGA cohort. (**d**) Overall survival curves and hazard tables for patients in Liu validation cohort. *P*-values were calculated using log rank test in all panels.

In primary cases, we observed a significant increase in survival in patients in Primary A compared to those in Primary B (Fig. 4a). Consistent with our observations from primary TCGA samples, a separate cohort of patients from whom primary samples were obtained^21^ also exhibited longer survival when ATP-dependent chaperones were expressed at lower levels (Fig. 4b). Surprisingly, we found that despite similar differences in PN gene expression, patients in Metastatic A had a significantly poorer survival than those in Metastatic B (Fig. 4c). This effect was also observed across metastatic patients divided into our original four subgroups (Supplementary Fig. 5b). Furthermore, an additional cohort of patients that donated metastatic samples exhibited a similar relationship between PN gene expression and survival to that observed in patients who donated metastatic TCGA samples (Fig. 4d and Supplementary Fig. 5b). Cox Proportional Hazard analysis confirmed that differences in survival between the sample groups persisted in both primary (*p* = 0.036) and metastatic (*p* = 0.002) groups when age, tumour stage at diagnosis and gender were taken into account (Supplementary Fig. 5c, d). Together, our data show that lower expression of ATP-dependent PN components within primary tumours is associated with better survival outcomes in CM patients, while a similar pattern of PN gene expression within metastasised tumours is associated with poorer survival.

To understand whether different clinical attributes could underlie differences in survival across CM patients, we assessed several melanoma characteristics that are dependent on the PN: pigmentation, rate of subsequent metastasis, drug response and tumour thickness across our patient sub-groups ^23,24^ wherever data was available. We found that in primary cohorts, samples in group A had higher pigmentation scores and lower subsequent metastasis than those in group B, as well as a trend towards reduced tumour thickness (Supplementary Fig. 6a–c). However, no difference in chemotherapy response was observed between our groups (Supplementary Fig. 6d).

Pigmentation was also increased in Metastatic group A compared to group B, but no difference in the other parameters measured was observed (Supplementary Fig. 6a–d). Lighter pigmentation was associated with better survival outcomes among our metastatic group (Supplementary Fig. 6f). However, this was not observed across primary samples (Supplementary Fig. 6e). In contrast, and as expected, levels of subsequent metastasis drove survival outcomes in patients from our primary sample group, as survival outcomes in individuals without subsequent metastasis were not significantly different between patients in our Primary A and B groups (Supplementary Fig. 6g).

Our data reveal that the differential expression of ATP-dependent PN components within primary (but not metastatic) CM tumours is associated with altered clinical features and survival outcomes within patients, most likely by influencing the likelihood of metastasis. These findings raise the possibility that alterations in the structure/strategy of the PN within tumours is a determinant of cancer progression. However, we cannot entirely rule out the possibility that differences in the expression of non-PN genes also contribute to the changes in clinical attributes and survival that we find across CM patients.

### The differential expression of proteostasis network genes is associated with increased expression of a core set of transcriptional regulators

Having established an association between PN gene expression signatures and clinical outcomes, we sought to identify the transcriptional regulators that might underlie different patterns of PN gene expression in primary and metastatic CM samples. To this purpose, we used RegEnrich^25^ to identify and rank factors that are most likely to account for differences in the expression of PN genes between groups.

**Figure 5:**
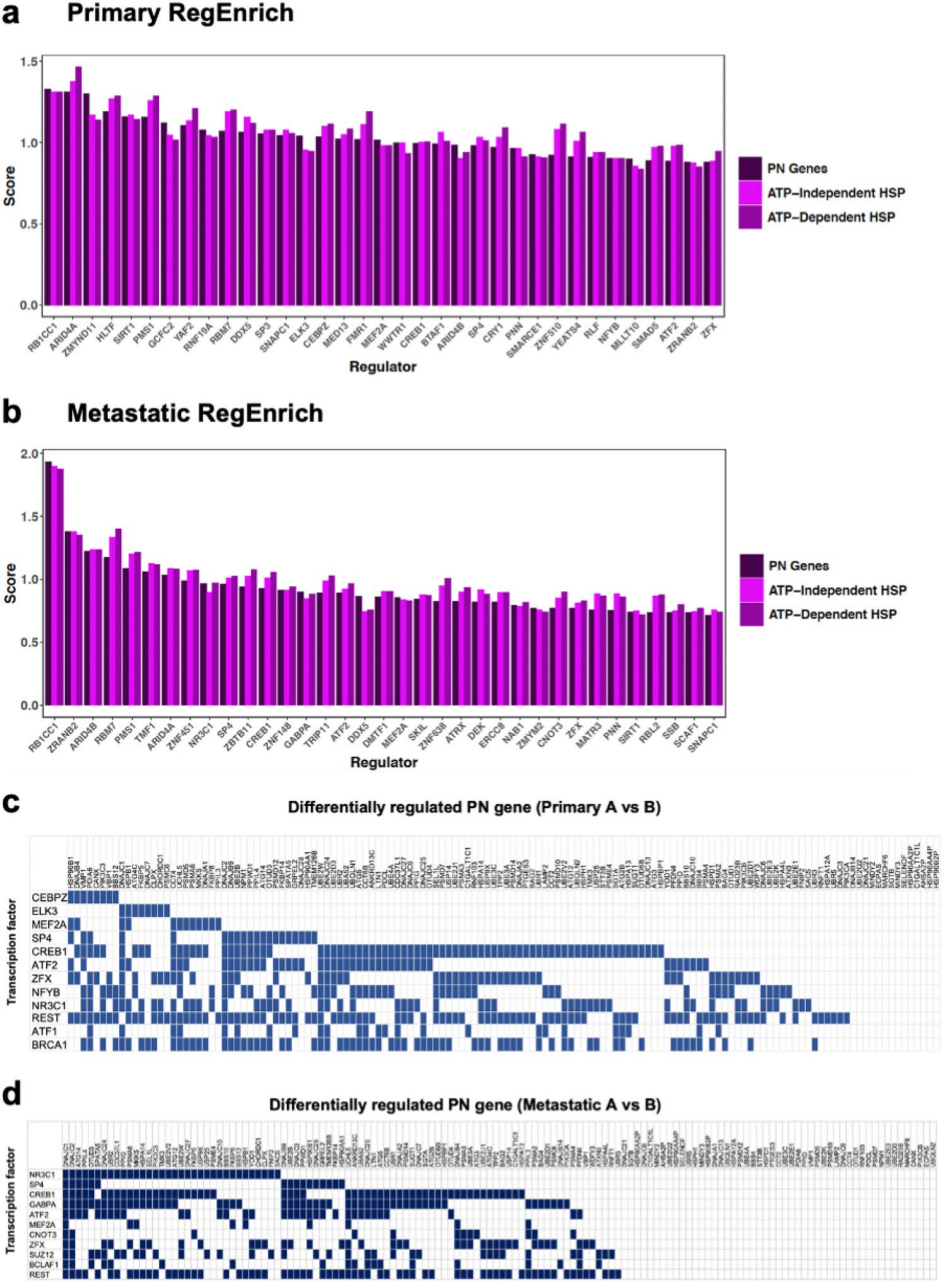
Distinct transcriptional regulators are associated with the differential expression of PN genes across CM samples: (**a**, **b**) RegEnrich scores of regulators identified from Proteostasis network (PN) gene expression changes in (**a**) primary and (**b**) metastatic TCGA cutaneous melanoma (CM) cohorts. (**c**, **d**) Matrices depicting transcription factors shown to directly bind differentially expressed PN genes by ChIP-seq, and highlighted as potential regulators by both RegEnrich and Enrichr in either (**c**) primary or (**d**) metastatic CM cohorts.

As expected, several common factors were identified among the top 20 potential regulators in primary and metastatic samples (Fig. 5a and b, Supplementary Fig. 7a). These included transcription factors (*RB1CC1, SP4, CREB1, ATF2, MEF2A, ZFX*), RNA binding proteins/helicases (*RBM7, DDX5, PNN*), a DNA mismatch repair factor (*PMS1*), a transcriptional coactivator (*TRIP11*) and transcriptional corepressors (*ARID4A, ARID4B, ZMYND11*) (Fig. 5a and b, Supplementary Tables 5 and 6).

Given that the most parsimonious driver of differences in PN gene expression between our groups is altered transcription factor activity, we used the Enrichr webtool^26,27^ to identify the RegEnrich candidate transcription factors whose targets are enriched among PN genes compared to the whole genome.

**Figure 6:**
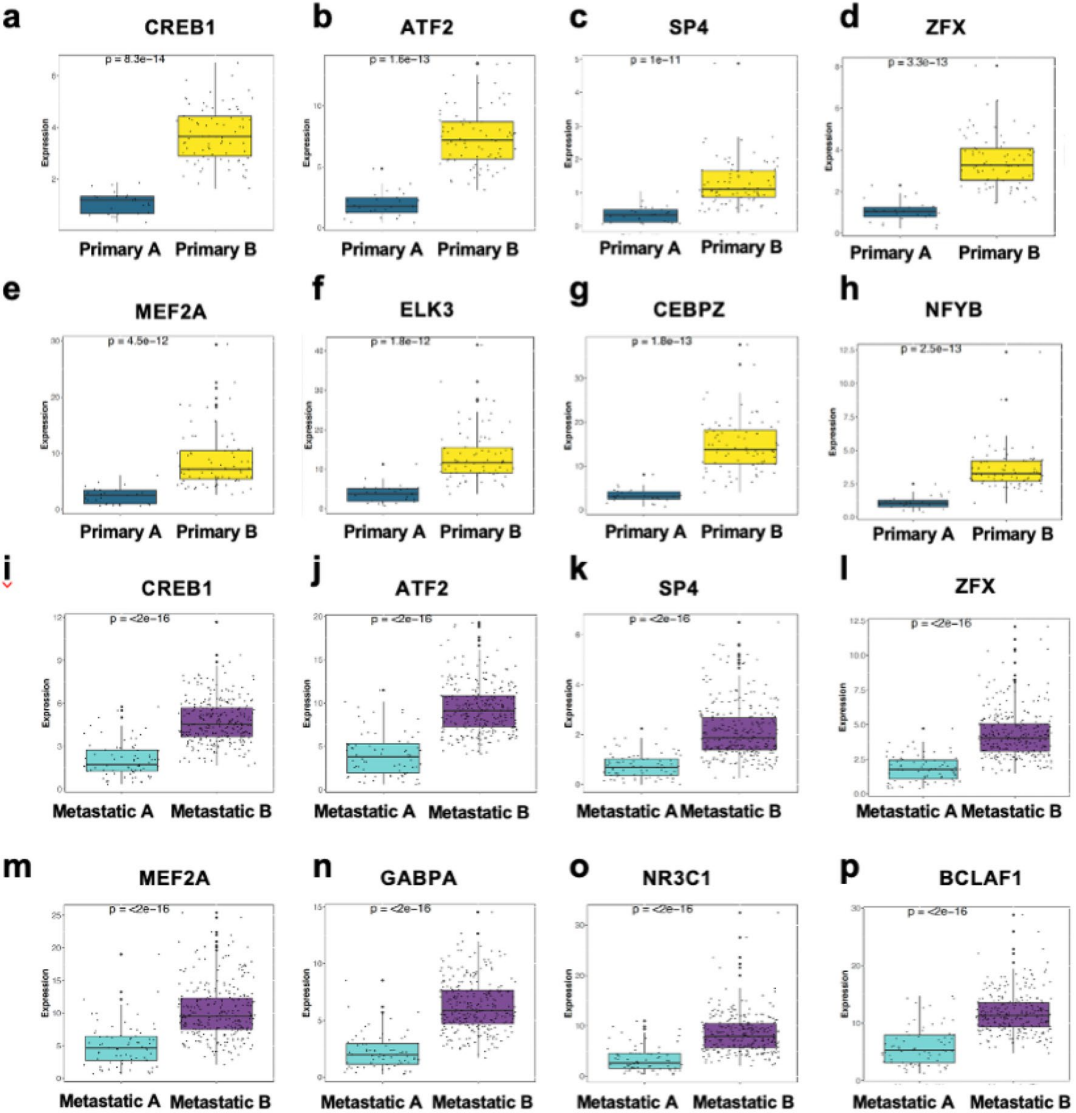
Differential expression of transcriptional regulators is associated with altered PN gene expression across CM samples. (**a**–**h**) Box plots showing the relative expression of transcription factors highlighted by RegEnrich and Enrichr as potential regulators of PN gene expression across primary groups A (n = 25) and B (n = 78). (**i**–**p**) Box plots showing the relative expression of transcription factors highlighted by RegEnrich and Enrichr as potential regulators of PN gene expression across metastatic groups A (n = 73) and B (n = 283). *P*-values were calculated using Student’s t-test. Boxes indicate the interquartile range (IQR), the upper whisker extends to the largest value that is less than (third quartile + (1.5 * IQR)). The lower whisker extends to the smallest value that is greater than (first quartile − (1.5 * IQR)).

We found that approximately 60 – 75% of differentially regulated PN genes in primary and metastatic samples are direct targets of the transcription factors CREB1, ATF2 and ZFX (Fig. 5c and d). Furthermore, the expression of these factors was increased 3- to 7-fold in group B samples from both primary and metastatic tissues (Fig. 6a–l). Similarly, other core regulators identified in primary samples (ELK3, CEBPZ and NFYB), metastatic samples (GABPA, NR3C1 and BLAF1) or both (SP4 and MEF2A) were elevated by a comparable level in group B (Fig. 6a–p). This elevated expression was not due to increased copy number variations within these genes (Fisher’s Exact Test *p* > 0.5) (Supplementary Fig. 7b) and is unlikely to be due to the differential incidence of mutations of these regulators across our samples (Supplementary Fig. 7c). Our data suggest that the expression of a common set of core transcription factors may underlie the shift towards either an ATP-dependent, or non-ATP dependent, proteostasis strategy across primary and metastatic CM samples.

## Discussion

Here, we have identified two distinct PN gene expression signatures within CM samples obtained from both primary and secondary cohorts. While there were differences in expression of genes across the PN, the most consistent and coherent differences were among molecular chaperone genes and subunits of the 19S and 20S proteasome. Among these changes, the most striking difference between individuals was a shift in the relative expression of ATP-dependent (e.g. HSP90) and non-ATP dependent (e.g. *HSPB1*) chaperones.

Both *HSPB1* and HSP90 have been reported to be up-regulated in many cancers, including lung, liver, pancreatic, breast and prostate cancer^28–31^. Increased levels of *HSPB1*, *HSPB2* and *HSPB5* having been shown to promote tumorigenesis through inhibition of protein aggregation and suppression of intrinsic and extrinsic pro-death factors^30^. In contrast, HSP90 has wide-ranging roles in the cell and promotes cancer through a multitude of mechanisms, including suppression of pro-apoptotic factors and facilitation of pro-oncogenic signalling^32^. In addition, extracellular HSP90 promotes tumour cell invasion, metastasis and angiogenesis ^24^. Therefore, our data suggest that primary and metastasised CM tumours can adopt different strategies, not just for maintaining the proteome, but also for promoting tumour formation and dispersal more generally.

Cancer cells have been shown to require high expression of various PN genes, including ATP-dependent and non-ATP dependent chaperones. Why then do primary and metastatic CM tumours exhibit such diversity in the expression of PN genes? A shift towards non-ATP dependent proteostasis strategies is also observed in the ageing brain and in Alzheimer’s, Huntington’s and Parkinson’s disease^33^, likely reflecting a beneficial adaptive response that protect cells during changes in metabolic and protein homeostasis^33^. Similarly, increased expression of sHSP coding genes is linked to impaired nutrient signalling, metabolic remodelling and reduced ATP levels, and is associated with protection against protein aggregation and environmental stress ^34,35^. HSPB1 has also been shown to protect melanoma cells against heat stress^36^. Interestingly, when mouse neural stem cells (NSPCs) differentiate into neural progenitor cells, the ATP-dependent chaperonin TRiC/CCT is down regulated and ATP-independent small heat shock proteins are upregulated^37^. This change is associated with increased protein aggregation and sequestration of misfolded proteins into inclusions^37^. Given that we observe a similar difference in the expression of sHSP and TRiC genes across CM patients, it is possible that some individuals with CM are more prone to protein aggregation than others due to differential remodelling of the PN. Therefore, the difference in PN gene expression in the CM samples may reflect altered metabolic homeostasis and reduced ATP availability in one set of tumours. Experiments to investigate levels of protein aggregation and metabolic markers in melanoma cells displaying these patterns of gene expression could confirm whether and how levels of protein aggregation differ between the two sample groups and whether differences in the PN are adaptive or maladaptive in primary and metastatic tumours.

Consistent with the role of HSP90 in promoting metastasis, we found that a shift towards higher levels of sHSPs and lower levels of HSP90 in primary CM tumours is associated with lower levels of regional metastasis and better survival outcomes. In contrast, higher sHSP expression and lower HSP90 expression within metastasised tumours was associated with poorer survival outcomes in patients. We propose that this may reflect the fact that once tumours have metastasised, patient death becomes more dependent on tumour growth and survival than further metastasis. As such, higher levels of sHSPs now become detrimental to individuals by promoting the survival and growth of metastasised tumours through enhanced proteostasis capacity and reduced apoptosis.

An alternative/complementary explanation for the differential association of PN gene expression with survival outcomes, is that tumour appearance (e.g. thickness, pigmentation), and therefore ease of detection, are dependent on the expression of PN genes. We did not detect any difference in tumour thickness between our two sample groups. However, we did find a clear association between PN gene expression and pigment levels in both primary and metastatic samples. Multiple PN components, including molecular chaperones and the ubiquitin proteasome system, are important for rates of melanin production^38,39^. As such, it is possible that the differential expression of PN genes leads to differences in melanogenesis and pigmentation between our patient groups. This could lead to melanomas being spotted by patients or clinicians at an earlier stage of development, thus leading to better response to chemotherapy, fewer subsequent metastases and improved survival.

While there was no difference in age between our patient groups, it is possible that differential PN gene expression reflects different stages of tumour development. Gene expression patterns alter in melanomas as they thicken and transition towards a more metastatic competent state^40^. Consistent with this, we found a trend towards increased thickness of the primary tumours across our different patient groups. Furthermore, while we aimed to correct gene expression signatures for differences in tumour purity based on immune cells, it is possible that the differences in PN gene expression reflect some other form of heterogeneity between tumours.

While differences in PN gene expression were highly consistent between primary and metastatic samples, they were not identical. For example, differential expression of subunits of the 19S proteasome regulatory particle was observed in both primary and metastatic samples. However, increased expression of 19S subunits was only observed across the metastatic cohort, whereas in the primary cohort, genes encoding 19S subunits were exclusively downregulated. The 19S regulatory particle of the proteasome recognizes and unfolds ubiquitin-tagged substrates and transfers them to the catalytic chamber of the 20S core^41^. Cancer cells are dependent on high levels of proteasome activity for growth and survival^42^, with proteasome inhibitors such as Bortezomib in clinical use to treat cancer^43^. However, counterintuitively, reduced expression of 19S subunits has also been shown to enhance resistance to proteosome inhibitors^44^, possibly through adaptive mechanisms that allow enhanced protein degradation and the maintenance of proteostasis capacity through alternative mechanisms. Therefore, it is possible that differences in the expression of proteasome subunits contribute to the different survival outcomes observed across our groups.

We have shown that the differential expression of PN genes across primary and metastatic CM samples can be explained by altered expression, and presumably activity, of a core set of transcription factors. Among these, *CREB* and *ATF* family transcription factors have been shown to promote melanoma^45^, while *MEF2A* and *ELK3* expression have been linked to other cancer types^46,47^. In addition, several of the potential regulators identified are associated with retinoblastoma or the retinoblastoma protein, RBP, which regulates cell proliferation. These include RB1CC1 (RB1 Inducible Coiled-Coil 1)^48^, *ARID4A* (AT-Rich Interaction Domain 4A, also known as Retinoblastoma-Binding Protein 1, *RBP1*)^49^ and *ARID4B* (AT-Rich Interaction Domain 4B, also known as Retinoblastoma-Binding Protein 1-Like 1, *RBP1L1*)^50^. Intriguingly, white adult survivors of retinoblastoma have a tenfold increased risk of developing and dying from melanoma compared with the general population^51^. Although the explanation for this is not known, it may be that the effect of retinoblastoma associated genes on proteostasis remodelling may partially explain the connection between retinoblastoma and melanoma.

Lastly, we also observed potential involvement of regulators including RNA binding and splicing factors, and DNA repair factors, suggesting that post-transcriptional mechanisms may also contribute to differential PN gene expression. Future experiments to demonstrate the relative contributions of these factors to PN gene expression in melanoma could identify new factors to target as part of future therapeutics. While our work has focused on the expression of PN components, the interaction networks formed between chaperones and their clients is also of fundamental importance to cancer^52^. At present, this has only been studied in the context of mitochondrial chaperones; however, further work to expand this to the PN more widely, may reveal distinct chaperone-client sub-networks within CM and other cancers.

Overall, our work has shown that the PN exhibits a high degree of transcriptional heterogeneity across CM samples such that at least two distinct patient groups can be demarcated based on PN gene expression. We also observe similar patterns in several other forms of cancer, suggesting that multiple PN sub-types may exist for many cancers. Given that targeting the PN (including small heat shock proteins, HSP90 and the proteasome) is a major focus of many cancer therapies in clinical use or development, our findings may have important ramifications for future cancer treatment strategies. For example, inhibiting HSP90 or the proteasome may be more effective in some CM patients than others, where targeting small heat shock proteins may be more beneficial. Similarly, deciding which components of the PN to target in primary or metastasised tumours may also be of relevance to ultimate patient outcomes.

## Methods

### Curation of a proteostasis network gene list

As cancer cell survival is strongly associated with protein folding and degradation, and pharmacologically targeting these pathways is a major focus of cancer therapeutics, we focused our attention on the core components of the PN that are related to these processes. To assemble our core PN list, we first used the Gene Ontology Resource^53,54^, the AmiGO^55^ web application and Uniprot^56^ to identify any genes with clearly defined primary roles in (i) folding of nascent proteins, (ii) refolding of non-native proteins and/or (iii) ubiquitination and degradation of terminally misfolded proteins by the ubiquitin proteasome system or autophagy. This initial gene list was then supplemented with genes listed in previous studies/reviews of chaperones, the UPS or autophagy^33,57,58^, before PN components with highly specific/limited targets, primary roles in the cell cycle, or poorly defined ancillary roles, were excluded. This resulted in 428 “core PN” genes, which were used for our subsequent analyses.

### Cancer samples

The TCGAbiolinks package^59^ was used to download FPKM normalised and raw RNA-sequencing expression data and clinical data aligned against the hg38 genome from the Muse pipeline from The Cancer Genome Atlas. Data were downloaded for 103 primary and 356 metastatic cutaneous melanoma (CM) samples and for all available samples of the other cancers listed in Supplementary Table 4. Expression data were log-transformed and tumour purity was accounted for by obtaining purity estimates from the TCGAbiolinks package and regressing the expression of each gene against tumour purity, applying the residuals for clustering of the CM samples. Exome-sequenced mutation and copy number data were obtained from cBioPortal (10.1158/2159-8290.cd-12-0095). Expression data for 36 primary CM samples from the Budden study were downloaded from the Gene Expression Omnibus https://www.ncbi.nlm.nih.gov/geo/query/acc.cgi?acc=GSE59455. Expression data for 72 metastatic CM samples from the Liu study were downloaded from cBIoportal https://www.cbioportal.org/study/summary?id=mel_dfci_2019.

### Normal samples

The normal skin tissue data used for the analyses described in this manuscript were obtained from the GTEx Portal on 1/11/2022. Both sun exposed and non-sun exposed skin samples were included. The Genotype-Tissue Expression (GTEx) Project was supported by the Common Fund of the Office of the Director of the National Institutes of Health, and by NCI, NHGRI, NHLBI, NIDA, NIMH, and NINDS. For comparison between normal and cancer samples we used GTEX and TCGA that had been normalised using Toil workflow software^60^.

### PN group identification and comparison

Expression data were adjusted for tumour purity^19^ before sample clusters were identified using the ComplexHeatmap^61^ package and Ward’s hierarchical agglomerative clustering method. The Student’s T test was used to compare expression of each PN gene between the two primary groups and between the two metastatic groups. Differential expression analysis using the DESeq2 R package^62^ was also carried out to compare gene expression between the groups. Genes were considered to have lower or higher expression in group A compared to group B if expression was found to be significantly lower or higher (adjusted *p* value < 0.1) in both analyses and if the fold change in expression between groups was < 0.8 or > 1.25.

### Comparison of normal and cancer samples

To compare expression in normal and cancer samples we used expression data from normal skin samples in the GTEX database and primary and metastatic CM samples from the TCGA database that had been uniformly processed and normalised and published by Wang et al^63^. Principal component analysis was performed using the factoextra R package.

### Mutational signature analysis

Mutational signature analysis was conducted using the deconstructSigs R package (10.1186/s13059-016-0893-4). Single base substitution signatures were obtained from COSMIC v3.2, and signatures present in > 5% of skin melanoma samples according to ICGC analysis (10.1038/s41586-020-1943-3) were included in further analysis. 16 samples with fewer than 50 somatic mutations were excluded from this analysis.

### Survival analysis

Clinical survival data was obtained from TCGA using TCGAbiolinks package. Survival plotes were drawn using ggsurvplot from the survminer R package as described at http://www.sthda.com/english/wiki/survminer-r-package-survival-data-analysis-and-visualization. Kaplan–Meier survival curves were calculated using the survfit function from the survival package as described in package documentation. P-values were calculated using the log-rank test^64^.

### Tumour microenvironment deconvolution from bulk RNA-seq data

The tumour microenvironment cell infiltration scores were calculated using the ConsensusTME R package^18^.

### Clinical data analysis

Clinical and demographic data, including incidence of subsequent metastasis, age at diagnosis,gender, survival and pigmentation, were downloaded from TCGA using TCGAbiolinks and from^65^ and distribution between clusters was analysed using Fisher’s Exact Test and Student’s t-test. Pigmentation of samples was classed as ‘low’ if the pigmentation score recorded in the TCGA clinical data was 0 or 1 and ‘high’ if their score was 2 or 3.

### Transcription factor targets and enrichment

Regulators that could explain differences in expression of PN genes were identified based on the scores allocated by Regenrich^66^ based on the expression of the regulators and their published targets in each cohort. Targets of transcription factors were identified using the Enrichr webtool^67^ using data from the CHEA 2022 Chip Seq database developed by Mayaan Lab and Encode TF ChIP-seq 2015 databases^68^. A gene was considered to be a target of a regulator if it was identified in any listed ChiP Seq experiment featured in the databases.

### Gene randomisation

Random genes were selected using the base R sample function without replacement.

### Data processing, analysis and code availability

All computational and statistical processing and analysis were carried out using the R programming language using the R Studio Integrated Development Environment. Data were processed using: the following R packages: matrixStats, data.table, dplyr, reshape, tidyverse, tidyr, loadRData, ggpubr, sva and the moveme function https://rdrr.io/github/mrdwab/SOfun/man/moveMe.html. Plots were created using ggplot2 and ggsurvplot. All code is available at https://github.com/ucbtrwe/Cutaneous_Melanoma.

### Ethics statement

All data employed in this study are publicly available and thus comply with ethical regulations, with approval and informed consent for collection and sharing already obtained by the respective consortia.

### Supplementary Information

Supplementary Information.

## Data Availability

Publicly available datasets analyzed in this study may be found here: TCGA: https://portal.gdc.cancer.gov/ (TCGA Genomics Data Commons Data Portal). Liu et al.: https://www.cbioportal.org/study/summary?id=mel_dfci_2019. Budden et al.: https://www.ncbi.nlm.nih.gov/geo/query/acc.cgi?acc=GSE59455
