# Supplementary material for: Distinct patterns of proteostasis network gene expression are associated with different prognoses in melanoma patients
Source: Sci Rep. 2024 Jan 2;14:198. doi: 10.1038/s41598-023-50640-0 (PMC10761826; doi:10.1038/s41598-023-50640-0)
Supplement: Supplementary file 1 — Supplementary Information. [file 41598_2023_50640_MOESM1_ESM.docx]

**Distinct patterns of proteostasis network gene expression are associated with different prognoses in melanoma patients**

Rachel Wellman, Daniel Jacobson, Maria Secrier and John Labbadia

Supplementary figures and tables


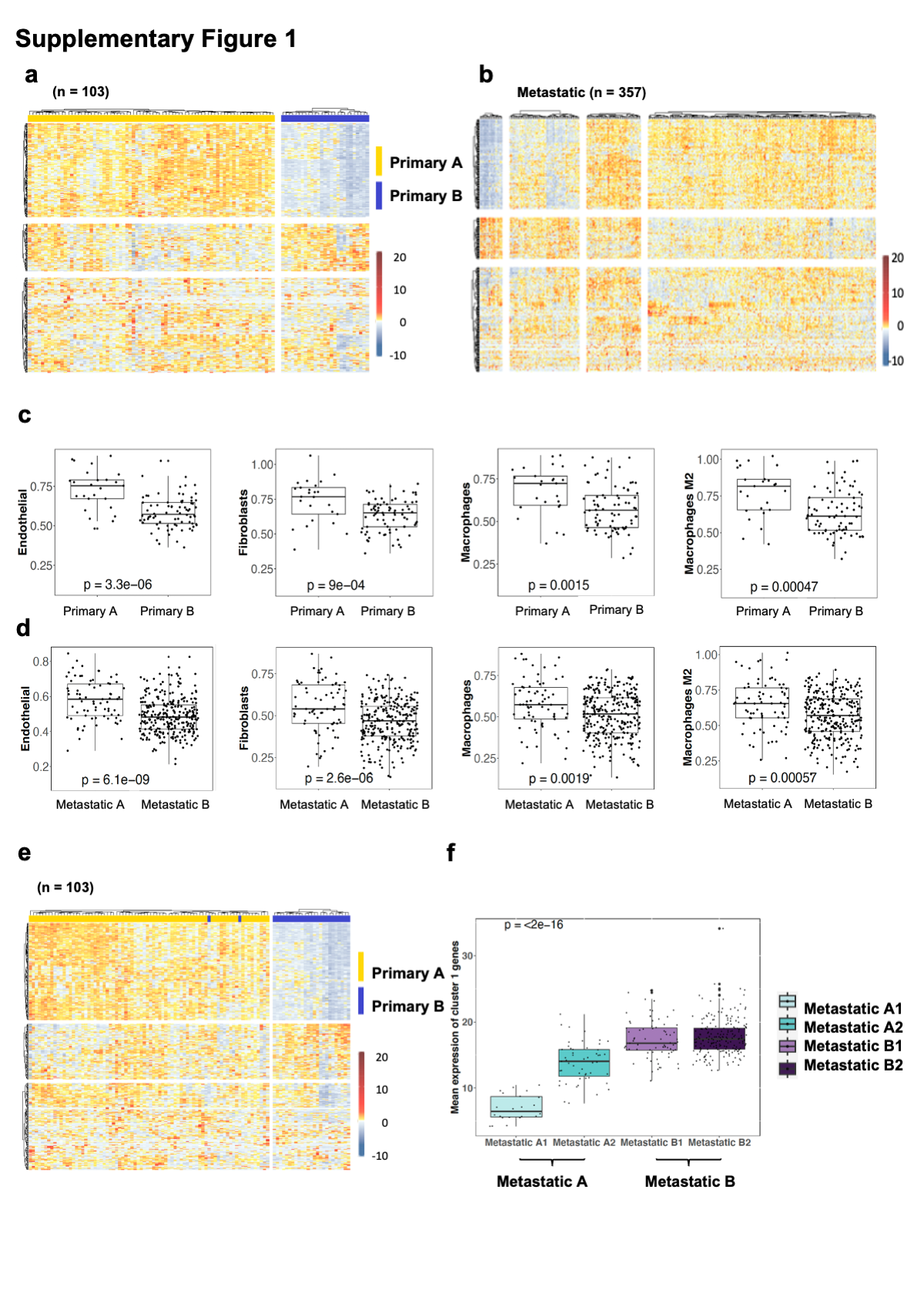


Figure 1: CM samples exhibit distinct PN gene expression patterns following correction for tumour purity

(a-b) PN gene expression in cutaneous melanoma (CM) (a) primary and (b) metastatic samples clustered using Ward’s hierarchical agglomerative clustering method. (c-d) Relative prevalence of endothelial cells, fibroblasts, macrophages and M2 macrophages in (c) primary and (d) metastatic CM sample groups. Primary A (n= 25), primary B (n = 78), metastatic A (n = 73) and metastatic B (n = 283). (e) PN gene expression in cutaneous melanoma (CM) primary samples following correction for tumour purity and clustering using Ward’s hierarchical agglomerative clustering method. (f) Mean expression of PN genes within cluster 1 of metastatic sample groups A1 (n = 22), A2 (n = 51), B1 (n = 75) and B2 (n = 208). In all cases, P-values were calculated using Student’s t-test. Boxes indicate the interquartile range (IQR), the upper whisker extends to the largest value that is less than (third quartile + (1.5 * IQR)). The lower whisker extends to the smallest value that is greater than (first quartile - (1.5 * IQR)).


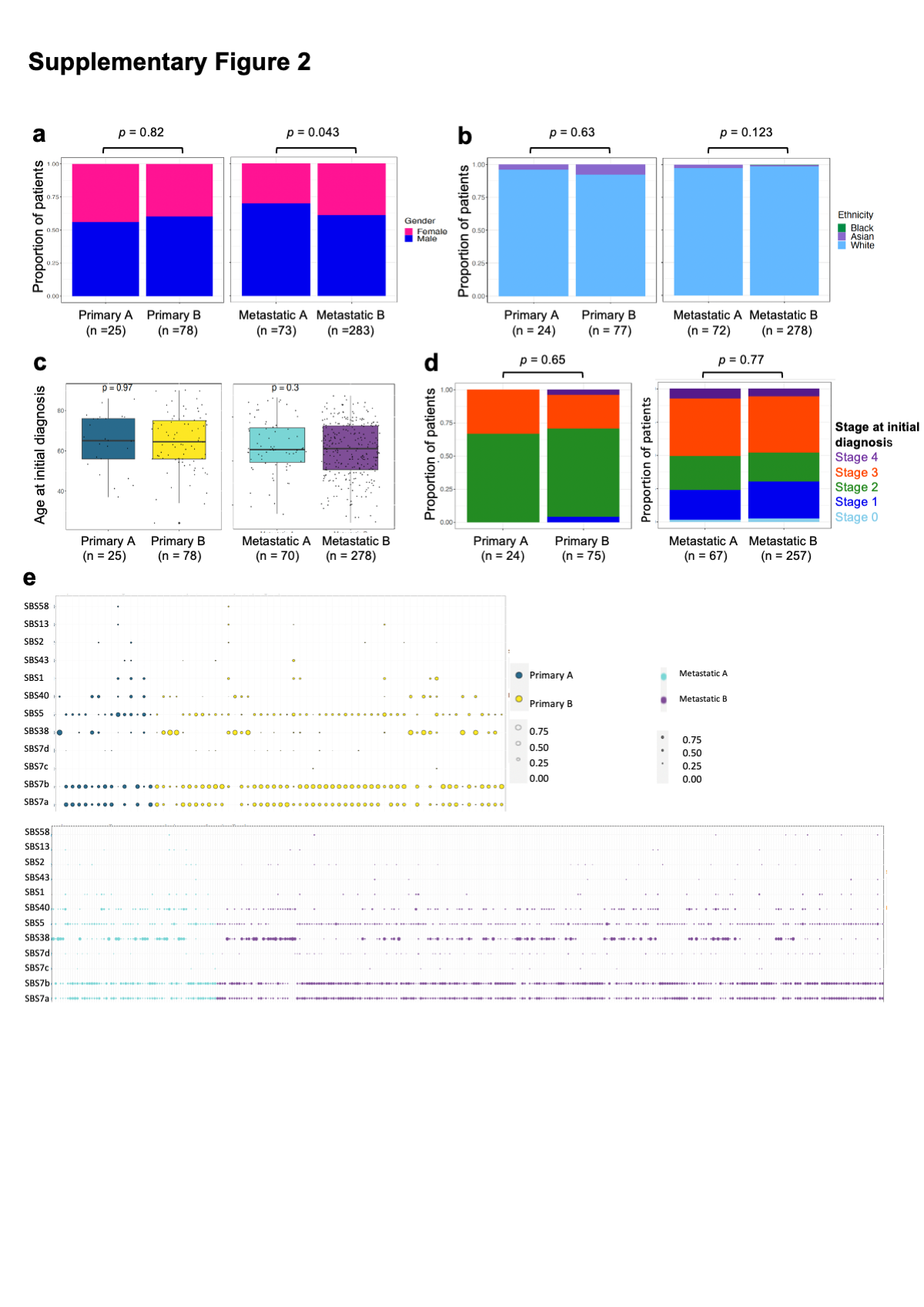


Supplementary Figure 2: Differences in PN gene expression are not associated with differences in demographic features or mutational signatures

(a-b) Proportion of cutaneous melanoma (CM) patients of (a) each gender or (b) different ethnicities within primary or metastatic groups A and B. (c) Mean age at diagnosis of patients in primary or metastatic groups A and B. Boxes indicate the interquartile range (IQR), the upper whisker extends to the largest value that is less than (third quartile + (1.5 * IQR)). The lower whisker extends to the smallest value that is greater than (first quartile - (1.5 * IQR)). (d) Proportion of CM patients at different stages of initial diagnosis in primary and metastatic groups A and B. (e) Balloon plots showing the relative contribution of different mutational signatures to tumours from primary and metastatic groups A and B. P-values were calculated using (a, b and d) Fisher’s Exact Test or (c) Student’s T-test.

**
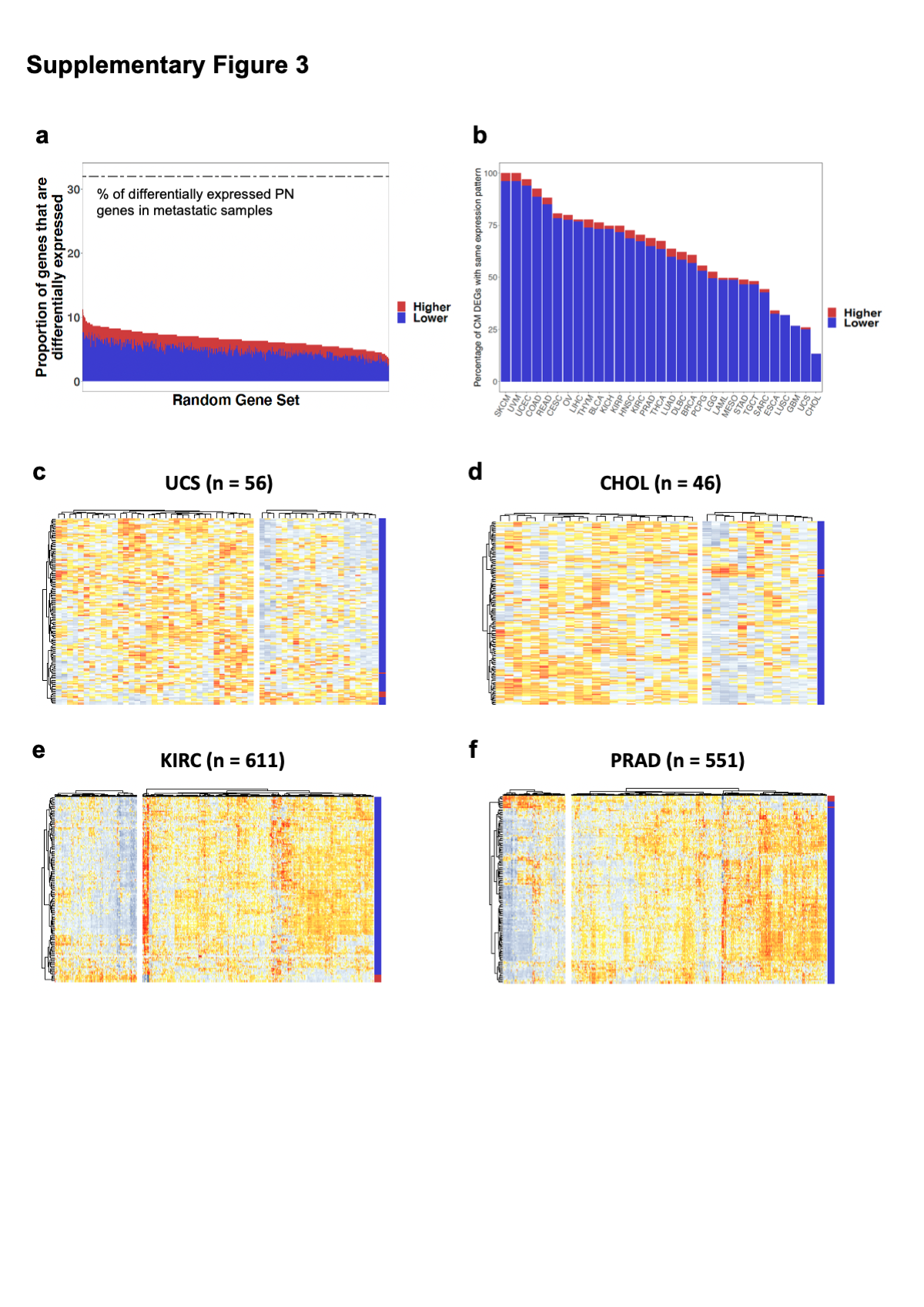
**

Supplementary Figure 3: Differential regulation of PN genes across CM samples shows low similarity with other cancers and is enriched changes compared to non-PN genes

(a) Proportion of random gene sets that were differentially expressed between cutaneous melanoma (CM) metastatic groups A and B (p-value < 0.05 calculated by Student’s T-test and DEseq2, adjusted p-value < 0.1 calculated by Benjamini Hochberg correction). (b) Percentage of genes that are differentially regulated PN between primary CM groups that are also differentially expressed in other cancers (p < 0.05 calculated by Student’s t-test adjusted p-value < 0.1 calculated by Benjamini Hochberg correction). (c-f) Heatmaps of PN genes differentially expressed in CM in (c) kidney renal clear cell carcinoma (KIRC) (d) prostate adenocarcinoma (PRAD), (e) uterine Carcinosarcoma (UCS) and (f) cholangiocarcinoma (CHOL).

**
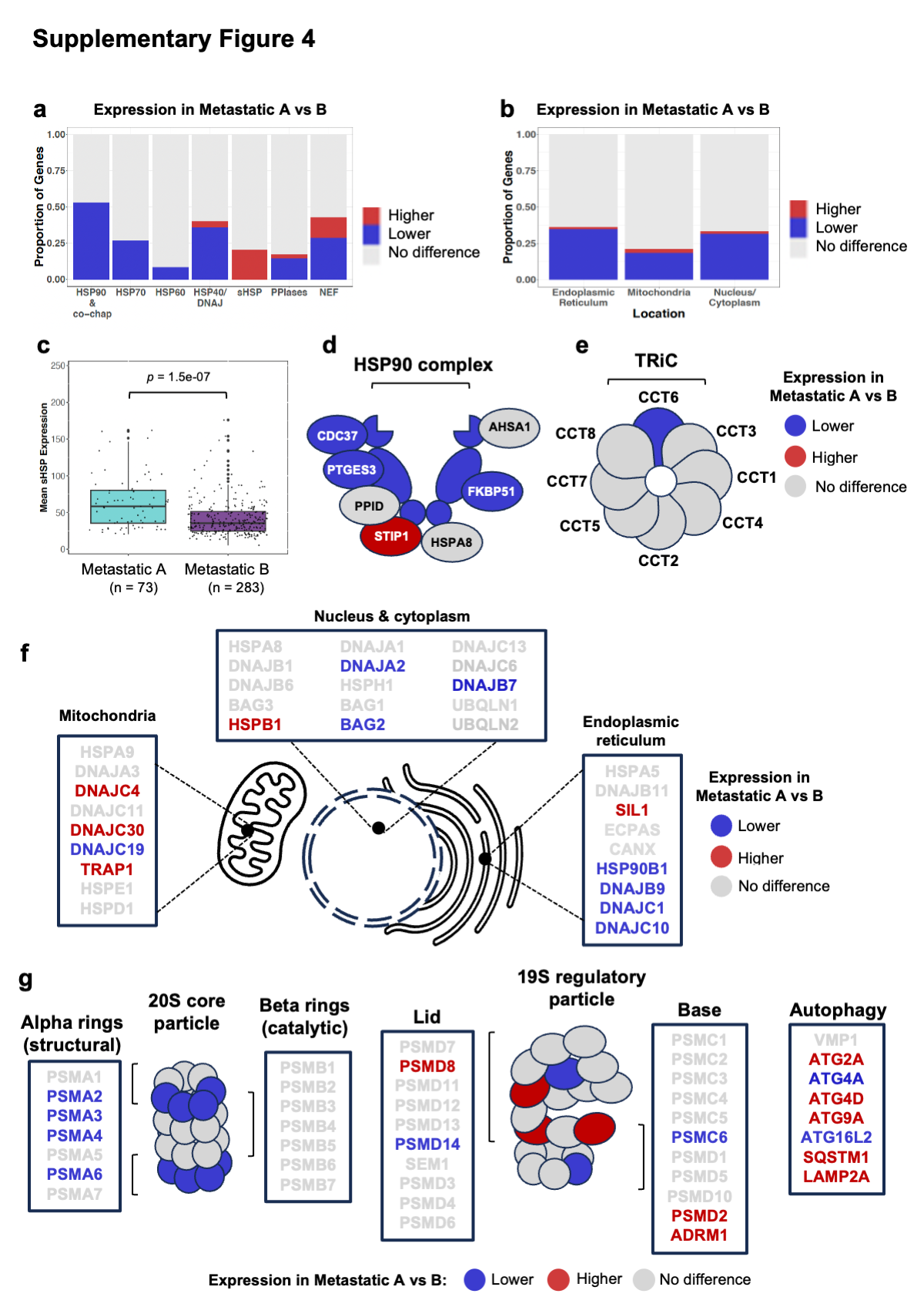
**

**Supplementary Figure 4:  Specific components of the sHSP, HSP90, HSP60, HSP70/DNAJ and proteasome systems are differentially expressed across metastatic CM samples**

(a) Proportion of genes within each Proteostasis network (PN) sub-group showing significantly altered expression between metastatic groups A and B (p-value < 0.05 calculated by Student’s T-test and DEseq2, adjusted p-value < 0.1 calculated by Benjamini Hochberg correction). (b) Proportion of PN genes within each sub-cellular compartment showing significantly altered expression between metastatic groups A and B (p-value < 0.05 calculated by Student’s T-test and DEseq2, adjusted p-value < 0.1 calculated by Benjamini Hochberg correction). (c) Mean expression of sHSP genes in metastatic sample groups. P-values were calculated using Student’s t-test. Boxes indicate the interquartile range (IQR), the upper whisker extends to the largest value that is less than (third quartile + (1.5 * IQR)). The lower whisker extends to the smallest value that is greater than (first quartile - (1.5 * IQR)). (d-g) Cartoons highlighting the PN components that exhibit differential expression between Primary A and B among (d) HSP90 and co-chaperones, (e) CCT/TRIC subunits (f) core chaperones and co-chaperones of sub-cellular compartments and (g) proteasome core and regulatory particle subunits and autophagy components.

**
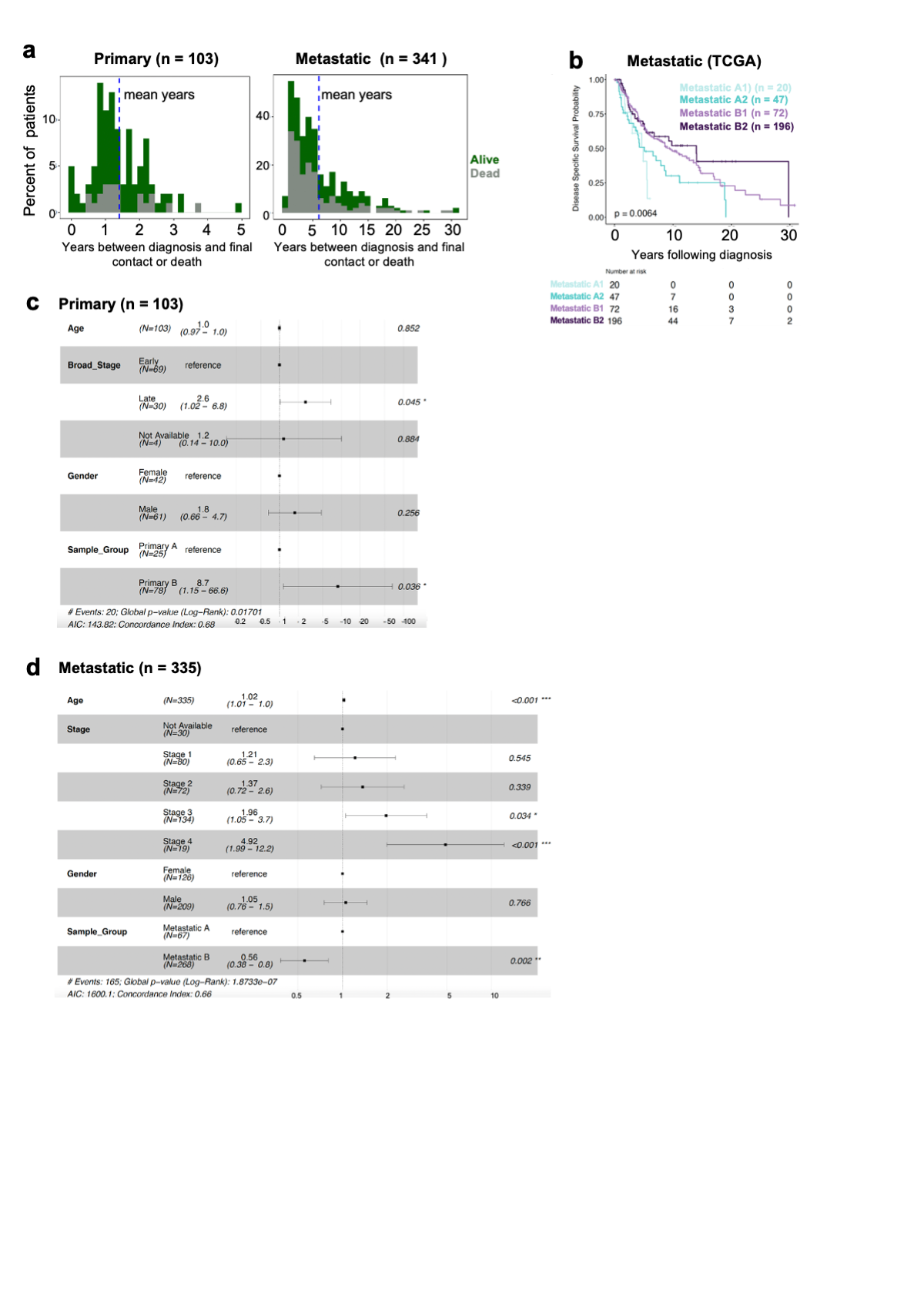
**

**Supplementary Figure 5: Stage at diagnosis, gender and stage of diseases do not fully explain survival differences between CM PN groups**

(a) Number of years between diagnosis and final contact or death, and mean number of years from diagnosis to final contact or death, in primary and metastatic cohorts. (b) Disease-specific survival curves for Metastatic A1, Metastatic A2, Metastatic B1 and Metastatic B2 patients. P values were calculated using log rank test. (c-d) Forrest plots showing the impact of age, clinical stage at diagnosis and gender on disease specific survival of individuals within (c) primary and (d) metastatic sample groups. P-values were calculated using Log-rank test.


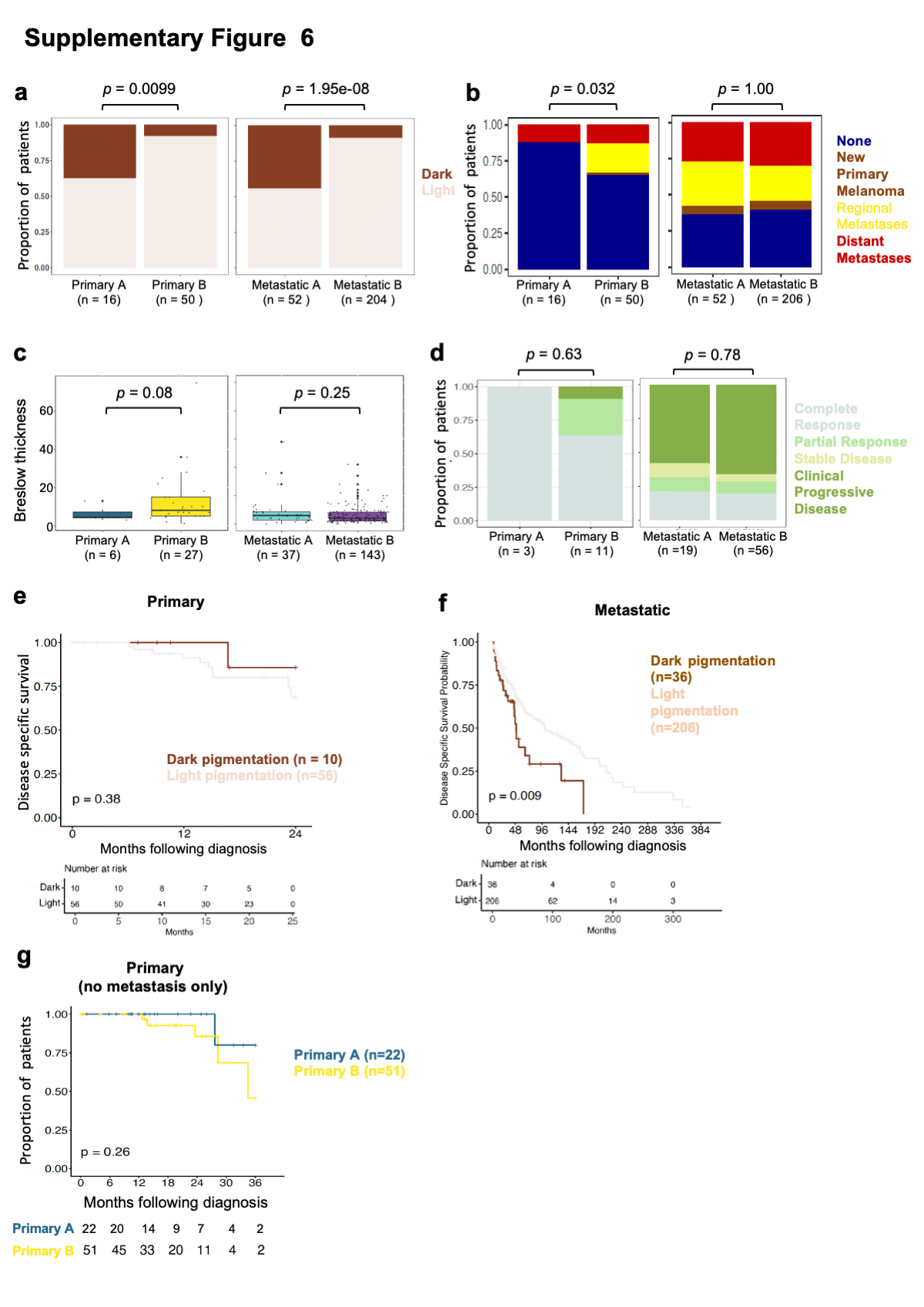


**Supplementary Figure 6: Survival differences between PN groups are associated with altered metastasis and tumour pigmentation**

(a-d) (a) Levels of pigmentation, (b) incidence of further metastasis, (c) tumour thickness and (d) response to chemotherapy across melanomas in primary and metastatic groups A and B. In (c), boxes indicate the interquartile range (IQR), the upper whisker extends to the largest value that is less than (third quartile + (1.5 * IQR)) and the lower whisker extends to the smallest value that is greater than (first quartile - (1.5 * IQR)). (e-f) Disease-specific survival curves by level of pigmentation in (e) primary or (f) metastatic patients. (g) Disease-specific survival curves for patients in primary groups A and B for whom no subsequent metastasis was recorded. P values were calculated using Fisher’s Exact Test (a, b and d), Student’s t-test (c) and Log rank test (e-g).

**
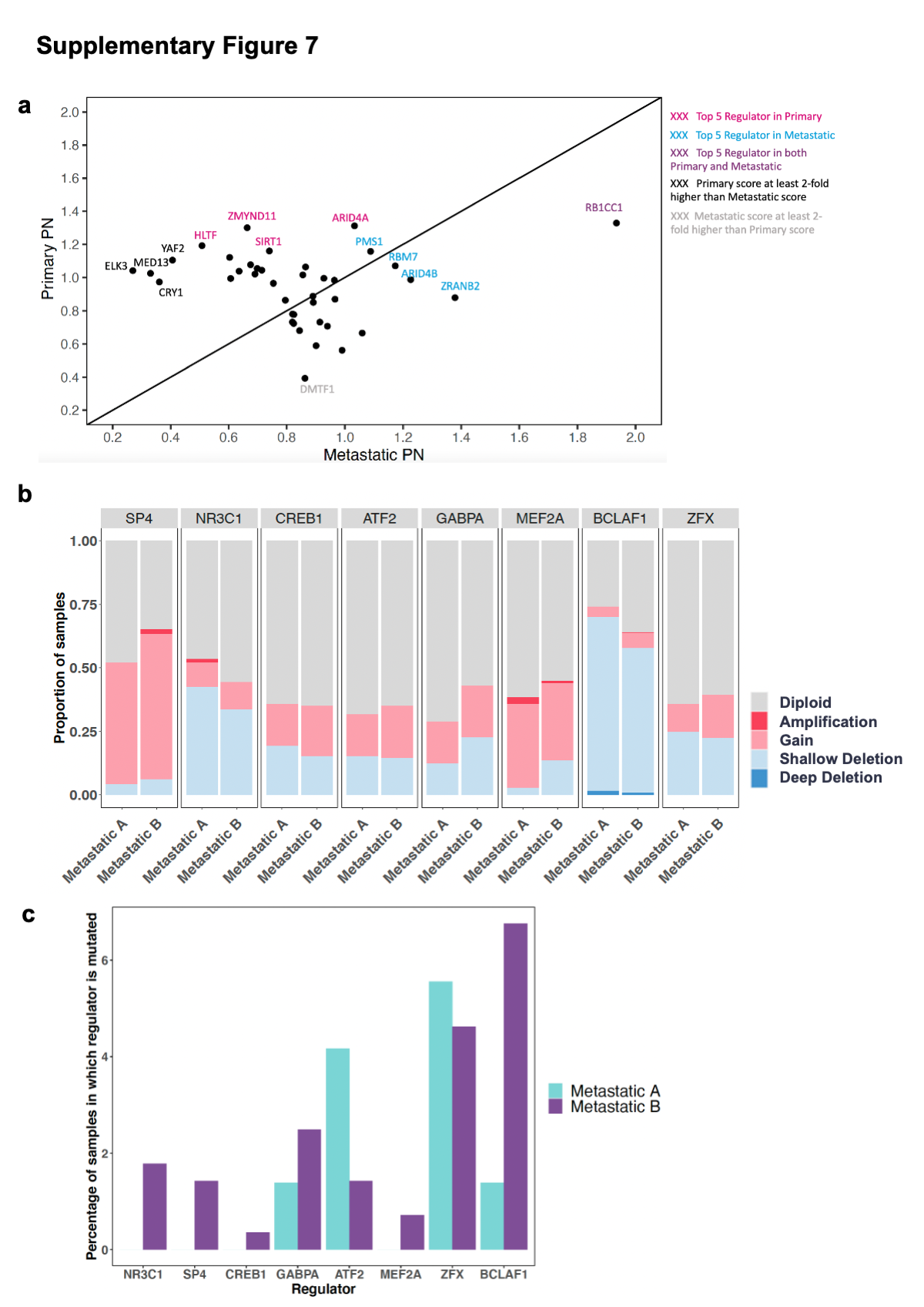
**

**Supplementary Figure 7: Specific transcriptional regulators are associated with differences in PN gene expression across CM samples but do not exhibit changes in copy number and exhibit low rates of mutation**

(a) Scatterplot of Regenrich scores for transcriptional regulators associated with differences in Proteostasis network gene expression across primary and metastatic cohorts. (b) Incidence of copy number variation of key regulators in metastatic groups A and B. (c) Incidence of non-synonymous mutations within key regulators across metastatic groups A and B.

**Supplementary Table 1: PN genes included in study**

| **Folding** | |
| --- | --- |
| **Gene Symbol** | **Gene Name** |
| AHSA1 | Activator of 90 kDa heat shock protein ATPase homolog 1 |
| AHSA2P | Putative activator of 90 kDa heat shock protein ATPase homolog 2 aka AHSA2 |
| AHSP | Alpha-hemoglobin-stabilizing protein |
| AIPL1 | Aryl-hydrocarbon-interacting protein-like 1 |
| ANKRD13C | Ankyrin repeat domain-containing protein 13C |
| BAG1 | BAG family molecular chaperone regulator 1 |
| BAG2 | BAG family molecular chaperone regulator 2 |
| BAG3 | BAG family molecular chaperone regulator 3 |
| BAG4 | BAG family molecular chaperone regulator 4 |
| BAG5 | BAG family molecular chaperone regulator 5 |
| BAG6 | Large proline-rich protein BAG6 |
| BBS10 | Bardet-Biedl syndrome 10 protein |
| BBS12 | Bardet-Biedl syndrome 12 protein |
| BBS4 | Bardet-Biedl syndrome 4 protein |
| BCAP31 | B-cell receptor-associated protein 31 |
| BCS1L | Mitochondrial chaperone BCS1 |
| C1GALT1C1 | C1GALT1-specific chaperone 1 |
| C1GALT1C1L | C1GALT1-specific chaperone 1-like protein |
| CALR | Calreticulin |
| CANX | Calnexin |
| CCT2 | T-complex protein 1 subunit beta |
| CCT3 | T-complex protein 1 subunit gamma |
| CCT4 | T-complex protein 1 subunit delta |
| CCT5 | T-complex protein 1 subunit epsilon |
| CCT6A | T-complex protein 1 subunit zeta |
| CCT6B | T-complex protein 1 subunit zeta-2 |
| CCT7 | T-complex protein 1 subunit eta |
| CCT8 | T-complex protein 1 subunit theta |
| CCT8L1P | Putative T-complex protein 1 subunit theta-like 1 |
| CCT8L2 | T-complex protein 1 subunit theta-like 2 |
| CDC37 | Hsp90 co-chaperone Cdc37 |
| CDC37L1 | Hsp90 co-chaperone Cdc37-like 1 |
| CHCHD4 | Mitochondrial intermembrane space import and assembly protein 40 |
| CHORDC1 | Cysteine and histidine-rich domain-containing protein 1 |
| CLGN | Calmegin |
| CLNS1A | Methylosome subunit pICln |
| CLPX | ATP-dependent Clp protease ATP-binding subunit clpX-like, mitochondrial |
| CLU | Clusterin |
| CNPY3 | Protein canopy homolog 3 |
| CRYAA | Alpha-crystallin A chain |
| CRYAB | Alpha-crystallin B chain |
| CYBC1 | Cytochrome b-245 chaperone 1 |
| DNAAF4 | Dynein assembly factor 4, axonemal |
| DNAJA1 | DnaJ homolog subfamily A member 1 |

| **Folding (continued)** | |
| --- | --- |
| **Gene Symbol** | **Gene Name** |
| DNAJA2 | DnaJ homolog subfamily A member 2 |
| DNAJA3 | DnaJ homolog subfamily A member 3, mitochondrial |
| DNAJA4 | DnaJ homolog subfamily A member 4 |
| DNAJB1 | DnaJ homolog subfamily B member 1 |
| DNAJB11 | DnaJ homolog subfamily B member 11 |
| DNAJB12 | DnaJ homolog subfamily B member 12 |
| DNAJB13 | DnaJ homolog subfamily B member 13 |
| DNAJB14 | DnaJ homolog subfamily B member 14 |
| DNAJB2 | DnaJ homolog subfamily B member 2 |
| DNAJB3 | DnaJ homolog subfamily B member 3 |
| DNAJB4 | DnaJ homolog subfamily B member 4 |
| DNAJB5 | DnaJ homolog subfamily B member 5 |
| DNAJB6 | DnaJ homolog subfamily B member 6 |
| DNAJB7 | DnaJ homolog subfamily B member 7 |
| DNAJB8 | DnaJ homolog subfamily B member 8 |
| DNAJB9 | DnaJ homolog subfamily B member 9 |
| DNAJC1 | DnaJ homolog subfamily C member 1 |
| DNAJC10 | DnaJ homolog subfamily C member 10 |
| DNAJC11 | DnaJ homolog subfamily C member 11 |
| DNAJC12 | DnaJ homolog subfamily C member 12 |
| DNAJC13 | DnaJ homolog subfamily C member 13 |
| DNAJC14 | DnaJ homolog subfamily C member 14 |
| DNAJC15 | DnaJ homolog subfamily C member 15 |
| DNAJC16 | DnaJ homolog subfamily C member 16 |
| DNAJC17 | DnaJ homolog subfamily C member 17 |
| DNAJC18 | DnaJ homolog subfamily C member 18 |
| DNAJC19 | Mitochondrial import inner membrane translocase subunit TIM14 |
| DNAJC2 | DnaJ homolog subfamily C member 2 |
| DNAJC21 | DnaJ homolog subfamily C member 21 |
| DNAJC22 | DnaJ homolog subfamily C member 22 |
| DNAJC24 | DnaJ homolog subfamily C member 24 |
| DNAJC25 | DnaJ homolog subfamily C member 25 |
| DNAJC27 | DnaJ homolog subfamily C member 27 |
| DNAJC28 | DnaJ homolog subfamily C member 28 |
| DNAJC3 | DnaJ homolog subfamily C member 3 |
| DNAJC30 | DnaJ homolog subfamily C member 30 |
| DNAJC4 | DnaJ homolog subfamily C member 4 |
| DNAJC5 | DnaJ homolog subfamily C member 5 |
| DNAJC5B | DnaJ homolog subfamily C member 5B |
| DNAJC5G | DnaJ homolog subfamily C member 5G |
| DNAJC6 | DnaJ homolog subfamily C member 6 |
| DNAJC7 | DnaJ homolog subfamily C member 7 =TPR2 |
| DNAJC8 | DnaJ homolog subfamily C member 8 |
| DNAJC9 | DnaJ homolog subfamily C member 9 |
| DNLZ | DNL-type zinc finger protein |
| ERP27 | Endoplasmic reticulum resident protein 27 |
| ERP29 | Endoplasmic reticulum resident protein 29 |
| ERP44 | Endoplasmic reticulum resident protein 44 |
| FKBP10 | Peptidyl-prolyl cis-trans isomerase FKBP10 |
| FKBP11 | Peptidyl-prolyl cis-trans isomerase FKBP11 |
| **Folding (continued)** | |
| **Gene Symbol** | **Gene Name** |
| FKBP14 | Peptidyl-prolyl cis-trans isomerase FKBP14 |
| FKBP15 | Peptidyl-prolyl cis-trans isomerase FKBP15 |
| FKBP1A | Peptidyl-prolyl cis-trans isomerase FKBP1A |
| FKBP1B | Peptidyl-prolyl cis-trans isomerase FKBP1B |
| FKBP1C | Peptidylprolyl isomerase |
| FKBP2 | Peptidyl-prolyl cis-trans isomerase FKBP2 |
| FKBP3 | Peptidyl-prolyl cis-trans isomerase FKBP3 |
| FKBP4 | Peptidyl-prolyl cis-trans isomerase FKBP4 |
| FKBP5 | Peptidyl-prolyl cis-trans isomerase FKBP5 |
| FKBP6 | Inactive peptidyl-prolyl cis-trans isomerase FKBP6 |
| FKBP7 | Peptidyl-prolyl cis-trans isomerase FKBP7 |
| FKBP8 | Peptidyl-prolyl cis-trans isomerase FKBP8 |
| FKBP9 | Peptidyl-prolyl cis-trans isomerase FKBP9 |
| FKBP9P1 | FKBP Prolyl Isomerase 9 Pseudogene 1 |
| FKBPL | Folliculin-interacting protein 1 |
| FNIP2 | Folliculin-interacting protein 2 |
| GAK | Cyclin-G-associated kinase |
| GRPEL1 | GrpE protein homolog 1, mitochondrial |
| GRPEL2 | GrpE protein homolog 2, mitochondrial |
| HOPX | Homeodomain-only protein |
| HSCB | Iron-sulfur cluster co-chaperone protein HscB |
| HSP90AA1 | Heat shock protein HSP 90-alpha |
| HSP90AA2P | Heat shock protein HSP 90-alpha A2 aka HSP90AA2 |
| HSP90AA4P | Putative heat shock protein HSP 90-alpha A4 |
| HSP90AA5P | Putative heat shock protein HSP 90-alpha A5 |
| HSP90AB1 | Heat shock protein HSP 90-beta |
| HSP90AB2P | Putative heat shock protein HSP 90-beta 2 |
| HSP90AB3P | Putative heat shock protein HSP 90-beta-3 |
| HSP90B1 | Endoplasmin |
| HSP90B2P | Putative endoplasmin-like protein |
| HSPA12A | Heat shock 70 kDa protein 12A |
| HSPA12B | Heat shock 70 kDa protein 12B |
| HSPA13 | Heat shock 70 kDa protein 13 |
| HSPA14 | Heat shock 70 kDa protein 14 |
| HSPA1A | Heat shock 70 kDa protein 1A |
| HSPA1B | Heat shock 70 kDa protein 1B |
| HSPA1L | Heat shock 70 kDa protein 1-like |
| HSPA2 | Heat shock-related 70 kDa protein 2 |
| HSPA4 | Heat shock 70 kDa protein 4 |
| HSPA4L | Heat shock 70 kDa protein 4L |
| HSPA5 | Endoplasmic reticulum chaperone BiP |
| HSPA6 | Heat shock 70 kDa protein 6 |
| HSPA7 | Putative heat shock 70 kDa protein 7 |
| HSPA8 | Heat shock cognate 71 kDa protein |
| HSPA9 | Stress-70 protein, mitochondrial |
| HSPB1 | Heat shock protein beta-1 |
| HSPB2 | Heat shock protein beta-2 |
| HSPB3 | Heat shock protein beta-3 |
| HSPB6 | Heat shock protein beta-6 |
| HSPB7 | Heat shock protein beta-7 |
| **Folding (continued)** | |
| **Gene Symbol** | **Gene Name** |
| HSPB8 | Heat shock protein beta-8 |
| HSPB9 | Heat shock protein beta-9 |
| HSPBP1 | Hsp70-binding protein 1 |
| HSPD1 | 60 kDa heat shock protein, mitochondrial |
| HSPE1 | 10 kDa heat shock protein, mitochondrial |
| HSPH1 | Heat shock protein 105 kDa |
| HYOU1 | Hypoxia up-regulated protein 1 |
| LRP2BP | LRP2-binding protein |
| LRPAP1 | Alpha-2-macroglobulin receptor-associated protein­­­­ |
| MKKS | McKusick-Kaufman/Bardet-Biedl syndromes putative chaperonin |
| NAA10 | N-Alpha-Acetyltransferase 10, NatA Catalytic Subunit |
| NPM1 | Nucleophosmin |
| P4HB | Protein disulfide-isomerase |
| PDCL | Phosducin-like protein |
| PDCL3 | Phosducin-like protein 3 |
| PDIA2 | Protein disulfide-isomerase A2 |
| PDIA3 | Protein disulfide-isomerase A3 |
| PDIA4 | Protein disulfide-isomerase A4 |
| PDIA5 | Protein disulfide-isomerase A5 |
| PDIA6 | Protein disulfide-isomerase A6 |
| PDRG1 | p53 and DNA damage-regulated protein 1 |
| PEX19 | Peroxisomal biogenesis factor 19 |
| PFDN1 | Prefoldin subunit 1 |
| PFDN2 | Prefoldin subunit 2 |
| PFDN4 | Prefoldin subunit 4 |
| PFDN5 | Prefoldin subunit 5 |
| PFDN6 | Prefoldin subunit 6 |
| PPIA | Peptidyl-prolyl cis-trans isomerase A |
| PPIAL4A | Peptidyl-prolyl cis-trans isomerase A-like 4A |
| PPIAL4C | Peptidyl-prolyl cis-trans isomerase A-like 4C |
| PPIAL4D | Peptidyl-prolyl cis-trans isomerase A-like 4D |
| PPIAL4E | Peptidyl-prolyl cis-trans isomerase A-like 4E |
| PPIAL4F | Peptidyl-prolyl cis-trans isomerase A-like 4F |
| PPIAL4G | Peptidyl-prolyl cis-trans isomerase A-like 4G |
| PPIAL4H | Peptidyl-prolyl cis-trans isomerase A-like 4H |
| PPIB | Peptidyl-prolyl cis-trans isomerase B |
| PPIC | Peptidyl-prolyl cis-trans isomerase C |
| PPID | Peptidyl-prolyl cis-trans isomerase D |
| PPIE | Peptidyl-prolyl cis-trans isomerase E |
| PPIF | Peptidyl-prolyl cis-trans isomerase F, mitochondrial |
| PPIG | Peptidyl-prolyl cis-trans isomerase G |
| PPIH | Peptidyl-prolyl cis-trans isomerase H |
| PPIL1 | Peptidyl-prolyl cis-trans isomerase-like 1 |
| PPIL3 | Peptidyl-prolyl cis-trans isomerase-like 3 |
| PPIL4 | Peptidyl-prolyl cis-trans isomerase-like 4 |
| PPWD1 | Peptidylprolyl isomerase domain and WD repeat-containing protein 1 |
| PTGES3 | Prostaglandin E synthase 3 |
| RCN3 | Reticulocalbin 3ulocalbin 3 |
| SACS | Sacsin |
| SELENOF | Selenoprotein F |
| **Folding (continued)** | |
| **Gene Symbol** | **Gene Name** |
| SERPINH1 | Serpin H1 |
| SGTA | Small glutamine-rich tetratricopeptide repeat-containing protein alpha |
| SGTB | Small glutamine-rich tetratricopeptide repeat-containing protein beta |
| SIL1 | Nucleotide exchange factor SIL1 |
| SNCA | Alpha-synuclein |
| SPATA5 | ATPase family protein 2 homolog |
| ST13 | Hsc70-interacting protein |
| STIP1 | Stress-induced-phosphoprotein 1 |
| TBCA | Tubulin-specific chaperone A |
| TBCC | Tubulin-specific chaperone C |
| TBCD | Tubulin-specific chaperone D |
| TCP1 | T-complex protein 1 subunit alpha |
| TIMM10 | Mitochondrial import inner membrane translocase subunit Tim10 |
| TIMM13 | Mitochondrial import inner membrane translocase subunit Tim13 |
| TIMM44 | Mitochondrial import inner membrane translocase subunit TIM44 |
| TIMM8A | Mitochondrial import inner membrane translocase subunit Tim8 A |
| TIMM8B | Mitochondrial import inner membrane translocase subunit Tim8 B |
| TIMM9 | Mitochondrial import inner membrane translocase subunit Tim9 |
| TMEM126B | Complex I assembly factor TMEM126B, mitochondrial |
| TMX3 | thioredoxin related transmembrane protein 3 |
| TOMM34 | Mitochondrial import receptor subunit TOM34 |
| TOR1A | Torsin-1A |
| TOR1B | Torsin-1B |
| TRAP1 | Heat shock protein 75 kDa, mitochondrial |
| TSACC | TSSK6-activating co-chaperone protein |
| TSC1 | Hamartin |
| TTC4 | Tetratricopeptide repeat protein 4 |
| UNC45B | Protein unc-45 homolog B |
| VBP1 | Prefoldin subunit 3 aka PFDN3 |

| **Ubiquitination** | |
| --- | --- |
| **Gene Symbol** | **Gene Name** |
| AMFR | E3 ubiquitin-protein ligase AMFR Autocrine motility factor receptor |
| CDC34 | Ubiquitin-conjugating enzyme E2 R1 |
| FBXO2 | F-box only protein 2 |
| FBXO6 | F-box only protein 6 |
| GET4 | Golgi to ER traffic protein 4 homolog |
| HUWE1 | E3 ubiquitin-protein ligase HUWE1 aka Tom1 |
| LRR1 | Leucine-rich repeat protein 1 |
| LRRC29 | Leucine-rich repeat-containing protein 29 |
| LRRC41 | Leucine-rich repeat-containing protein 41 |
| LTN1 | E3 ubiquitin-protein ligase listerin |
| MARCHF6 | E3 ubiquitin-protein ligase MARCHF6 |
| PRKN | E3 ubiquitin-protein ligase parkin |
| RBX1 | E3 ubiquitin-protein ligase RBX1 |
| RNF103 | E3 ubiquitin-protein ligase RNF103 |
| RNF126 | E3 ubiquitin-protein ligase RNF126 |
| RNF185 | E3 ubiquitin-protein ligase RNF185 |
| RNF5 | E3 ubiquitin-protein ligase RNF5 |
| RNFT1 | E3 ubiquitin-protein ligase RNFT1 |
| RPS27A | Ubiquitin-40S ribosomal protein S27a |
| SEL1L | Protein sel-1 homolog 1 |
| SELENOS | Selenoprotein S |
| STT3B | Dolichyl-diphosphooligosaccharide-protein glycosyltransferase subunit STT3B |
| STUB1 | E3 ubiquitin-protein ligase CHIP |
| SUGT1 | Protein SGT1 homolog |
| SYVN1 | E3 ubiquitin-protein ligase synoviolin |
| TRAF2 | TNF receptor-associated factor 2 |
| TRAF3 | TNF receptor-associated factor 3 |
| TRIM11 | E3 ubiquitin-protein ligase TRIM11 |
| TRIM13 | E3 ubiquitin-protein ligase TRIM13 |
| UBA1 | Ubiquitin-like modifier-activating enzyme 1 |
| UBA52 | Ubiquitin-60S ribosomal protein L40 |
| UBE2A | Ubiquitin-conjugating enzyme E2 A |
| UBE2B | Ubiquitin-conjugating enzyme E2 B |
| UBE2C | Ubiquitin-conjugating enzyme E2 C |
| UBE2D1 | Ubiquitin-conjugating enzyme E2 D1 |
| UBE2D2 | Ubiquitin-conjugating enzyme E2 D2 |
| UBE2D3 | Ubiquitin-conjugating enzyme E2 D3 |
| UBE2D4 | Ubiquitin-conjugating enzyme E2 D4 |
| UBE2E1 | Ubiquitin-conjugating enzyme E2 E1 |
| UBE2F | Ubiquitin-conjugating enzyme E2 F |
| UBE2G1 | Ubiquitin-conjugating enzyme E2 G1 |
| UBE2G2 | Ubiquitin-conjugating enzyme E2 G2 |
| UBE2H | Ubiquitin-conjugating enzyme E2 H |
| UBE2J1 | Ubiquitin-conjugating enzyme E2 J1 |
| UBE2J2 | Ubiquitin-conjugating enzyme E2 J2 |
| UBE2K | Ubiquitin-conjugating enzyme E2 K |
| UBE2L3 | Ubiquitin-conjugating enzyme E2 L3 |
| UBE2L5 | Ubiquitin-conjugating enzyme E2 L5 |
| UBE2L6 | Ubiquitin/ISG15-conjugating enzyme E2 L6 |
| UBE2M | NEDD8-conjugating enzyme Ubc12 |
| UBE2N | Ubiquitin-conjugating enzyme E2 N |
| UBE2NL | Putative ubiquitin-conjugating enzyme E2 N-like |
| UBE2O | "(E3-independent) E2 ubiquitin-conjugating enzyme" |
| UBE2Q1 | Ubiquitin-conjugating enzyme E2 Q1 |
| UBE2Q2 | Ubiquitin-conjugating enzyme E2 Q2 |
| **Ubiquitination (continued)** | |
| **Gene Symbol** | **Gene Name** |
| UBE2QL1 | Ubiquitin-conjugating enzyme E2Q-like protein 1 |
| UBE2R2 | Ubiquitin-conjugating enzyme E2 R2 |
| UBE2S | Ubiquitin-conjugating enzyme E2 S |
| UBE2U | Ubiquitin-conjugating enzyme E2 U |
| UBE2V1 | Ubiquitin-conjugating enzyme E2 variant 1 |
| UBE2V2 | Ubiquitin-conjugating enzyme E2 variant 2 |
| UBE2W | Ubiquitin-conjugating enzyme E2 W |
| UBE2Z | Ubiquitin-conjugating enzyme E2 Z |
| UBE3A | Ubiquitin-protein ligase E3A aka E6-AP |
| UBE3B | Ubiquitin-protein ligase E3B |
| UBE3C | Ubiquitin-protein ligase E3C aka Hul5 |
| UBE3D | E3 ubiquitin-protein ligase E3D |
| UBE4A | Ubiquitin conjugation factor E4 A |
| UBE4B | Ubiquitin conjugation factor E4 B |
| UBR1 | E3 ubiquitin-protein ligase UBR1 |
| UBR2 | E3 ubiquitin-protein ligase UBR2 |
| UBR3 | E3 ubiquitin-protein ligase UBR3 |
| UBR4 | E3 ubiquitin-protein ligase UBR4 |
| UBR5 | E3 ubiquitin-protein ligase UBR5 |
| UBR7 | Putative E3 ubiquitin-protein ligase UBR7 |

| **Deubiquitination** | |
| --- | --- |
| **Gene Symbol** | **Gene Name** |
| ATXN3 | Ataxin-3 |
| MINDY1 | Ubiquitin carboxyl-terminal hydrolase MINDY-1 |
| MINDY2 | Ubiquitin carboxyl-terminal hydrolase MINDY-2 |
| MINDY3 | Ubiquitin carboxyl-terminal hydrolase MINDY-3 |
| MINDY4 | Probable ubiquitin carboxyl-terminal hydrolase MINDY-4 |
| OTUB1 | Ubiquitin thioesterase OTUB1 |
| OTUB2 | Ubiquitin thioesterase OTUB2 |
| OTUD1 | OTU domain-containing protein 1 |
| OTUD3 | OTU domain-containing protein 3 |
| OTUD4 | OTU domain-containing protein 4 |
| OTUD5 | OTU domain-containing protein 5 |
| OTUD6A | OTU domain-containing protein 6A |
| OTUD6B | Deubiquitinase OTUD6B |
| OTUD7A | OTU domain-containing protein 7A |
| UCHL1 | Ubiquitin carboxyl-terminal hydrolase isozyme L1 |
| UCHL3 | Ubiquitin carboxyl-terminal hydrolase isozyme L3 |
| UCHL5 | Ubiquitin carboxyl-terminal hydrolase isozyme L5 aka CGI-70, INO80R, UCH37 |
| USP14 | Ubiquitin carboxyl-terminal hydrolase 14 |
| USP19 | Ubiquitin carboxyl-terminal hydrolase 19 |
| USP25 | Ubiquitin carboxyl-terminal hydrolase 25 |
| USP26 | Ubiquitin carboxyl-terminal hydrolase 26 |
| USP4 | Ubiquitin carboxyl-terminal hydrolase 4 |
| USP9X | Probable ubiquitin carboxyl-terminal hydrolase FAF-X |
| YOD1 | Ubiquitin thioesterase OTU1 |

| **Degradation** | |
| --- | --- |
| **Gene Symbol** | **Gene Name** |
| ADRM1 | Proteasomal ubiquitin receptor ADRM1 |
| ECPAS | Proteasome adapter and scaffold protein ECM29 |
| NFE2L1 | Endoplasmic reticulum membrane sensor NFE2L1 |
| PAAF1 | Proteasomal ATPase-associated factor 1 |
| POMP | Proteasome maturation protein |
| PSMA1 | Proteasome subunit alpha type-1 |
| PSMA2 | Proteasome subunit alpha type-2 |
| PSMA3 | Proteasome subunit alpha type-3 |
| PSMA4 | Proteasome subunit alpha type-4 |
| PSMA5 | Proteasome subunit alpha type-5 |
| PSMA6 | Proteasome subunit alpha type-6 |
| PSMA7 | Proteasome subunit alpha type-7 |
| PSMA8 | Proteasome subunit alpha-type 8 |
| PSMB1 | Proteasome subunit beta type-1 |
| PSMB10 | Proteasome subunit beta type-10 |
| PSMB11 | Proteasome subunit beta type-11 |
| PSMB2 | Proteasome subunit beta type-2 |
| PSMB3 | Proteasome subunit beta type-3 |
| PSMB4 | Proteasome subunit beta type-4 |
| PSMB5 | Proteasome subunit beta type-5 |
| PSMB6 | Proteasome subunit beta type-6 |
| PSMB7 | Proteasome subunit beta type-7 |
| PSMB8 | Proteasome subunit beta type-8 |
| PSMB9 | Proteasome subunit beta type-9 |
| PSMC1 | 26S proteasome regulatory subunit 4 |
| PSMC2 | 26S proteasome regulatory subunit 7 |
| PSMC3 | 26S proteasome regulatory subunit 6A |
| PSMC4 | 26S proteasome regulatory subunit 6B |
| PSMC5 | 26S proteasome regulatory subunit 8 |
| PSMC6 | 26S proteasome regulatory subunit 10B |
| PSMD1 | 26S proteasome non-ATPase regulatory subunit 1 |
| PSMD10 | 26S proteasome non-ATPase regulatory subunit 10 |
| PSMD11 | 26S proteasome non-ATPase regulatory subunit 11 |
| PSMD12 | 26S proteasome non-ATPase regulatory subunit 12 |
| PSMD13 | 26S proteasome non-ATPase regulatory subunit 13 |
| PSMD14 | 26S proteasome non-ATPase regulatory subunit 14 |
| PSMD2 | 26S proteasome non-ATPase regulatory subunit 2 |
| PSMD3 | 26S proteasome non-ATPase regulatory subunit 3 |
| PSMD4 | 26S proteasome non-ATPase regulatory subunit 4 |
| PSMD5 | 26S proteasome non-ATPase regulatory subunit 5 |
| PSMD6 | 26S proteasome non-ATPase regulatory subunit 6 |
| PSMD7 | 26S proteasome non-ATPase regulatory subunit 7 |
| PSMD8 | 26S proteasome non-ATPase regulatory subunit 8 |
| PSMD9 | 26S proteasome non-ATPase regulatory subunit 9 |
| PSME1 | Proteasome activator complex subunit 1 |
| PSME2 | Proteasome activator complex subunit 2 |
| PSME3 | Proteasome activator complex subunit 3 |
| PSME4 | Proteasome activator complex subunit 4 |
| PSMF1 | Proteasome inhibitor PI31 subunit |
| PSMG1 | Proteasome assembly chaperone 1 |
| PSMG2 | Proteasome assembly chaperone 2 |
| PSMG3 | Proteasome assembly chaperone 3 |
| PSMG4 | Proteasome assembly chaperone 4 |
| RAD23A | UV excision repair protein RAD23 homolog A |
| RAD23B | UV excision repair protein RAD23 homolog B |

| **Degradation (continued)** | |
| --- | --- |
| **Gene Symbol** | **Gene Name** |
| SEM1 | 26S proteasome complex subunit SEM1 |
| TPP2 | Tripeptidyl-peptidase 2 |
| UBQLN1 | Ubiquilin-1 |
| UBQLN2 | Ubiquilin-2 |
| UBQLN4 | Ubiquilin-4 |
| ZSWIM2 | E3 ubiquitin-protein ligase ZSWIM2 |

| **Autophagy** | |
| --- | --- |
| **Gene Symbol** | **Gene Name** |
| ATG10 | Ubiquitin-like-conjugating enzyme ATG10 |
| ATG101 | Autophagy-related protein 101 |
| ATG12 | Ubiquitin-like protein ATG12 |
| ATG13 | Autophagy-related protein 13 |
| ATG14 | Beclin 1-associated autophagy-related key regulator |
| ATG16L1 | Autophagy-related protein 16-1 |
| ATG16L2 | Autophagy-related protein 16-2 |
| ATG2A | Autophagy-related protein 2 homolog A |
| ATG2B | Autophagy-related protein 2 homolog B |
| ATG3 | Ubiquitin-like-conjugating enzyme ATG3 |
| ATG4A | Cysteine protease ATG4A |
| ATG4B | Cysteine protease ATG4B |
| ATG4C | Cysteine protease ATG4C |
| ATG4D | Cysteine protease ATG4D |
| ATG5 | Autophagy protein 5 |
| ATG7 | Ubiquitin-like modifier-activating enzyme ATG7 |
| ATG9A | Autophagy-related protein 9A |
| ATG9B | Autophagy-related protein 9B |
| ATXN3L | Ataxin-3-like protein |
| BECN1 | Beclin-1 aka ATG6 |
| BECN2 | Beclin-2 |
| GABARAP | Gamma-aminobutyric acid receptor-associated protein |
| GABARAPL1 | Gamma-aminobutyric acid receptor-associated protein-like 1 |
| GABARAPL2 | Gamma-aminobutyric acid receptor-associated protein-like 2 |
| HDAC6 | Histone deacetylase 6 |
| LAMP2 | Lysosome-associated membrane glycoprotein 2 |
| MAP1LC3A | Microtubule-associated proteins 1A/1B light chain 3A |
| MAP1LC3B | Microtubule-associated proteins 1A/1B light chain 3B |
| MAP1LC3B2 | Microtubule-associated proteins 1A/1B light chain 3 beta 2 |
| MAP1LC3C | Microtubule-associated proteins 1A/1B light chain 3C |
| PIK3C2B | Phosphatidylinositol 4-phosphate 3-kinase C2 domain-containing subunit beta |
| PIK3C3 | Phosphatidylinositol 3-kinase catalytic subunit type 3 |
| PIK3CA | Phosphatidylinositol 4,5-bisphosphate 3-kinase catalytic subunit alpha isoform |
| PIK3CB | Phosphatidylinositol 4,5-bisphosphate 3-kinase catalytic subunit beta isoform |
| PIK3R2 | Phosphatidylinositol 3-kinase regulatory subunit beta |
| PIK3R4 | Phosphoinositide 3-kinase regulatory subunit 4 |
| SQSTM1 | Sequestosome-1 |
| ULK1 | Serine/threonine-protein kinase ULK1 aka ATG1 |
| VMP1 | Vacuole membrane protein 1 |
| WDFY3 | WD repeat and FYVE domain-containing protein 3 |
| WDR45 | WD repeat domain phosphoinositide-interacting protein 4 |
| WDR45B | WD repeat domain phosphoinositide-interacting protein 3 |

**Supplementary Table 2: Significance of difference in levels of immune cell infiltration**

| **Immune Cell Type** | **Primary p.value** | **Primary Significance** | **Metastatic p.value** | **Metastatic Significance** |
| --- | --- | --- | --- | --- |
| Endothelial | 0.0000033 | **** | 6.1E-09 | **** |
| Fibroblasts | 0.0009 | *** | 0.0000026 | **** |
| Macrophages M2 | 0.00047 | *** | 0.00057 | *** |
| Macrophages | 0.0015 | ** | 0.0019 | ** |
| Neutrophils | 0.0064 | ** | 0.0093 | ** |
| Eosinophils | 0.008 | ** | 0.008 | ** |
| Monocytes | 0.0041 | ** | 0.012 | * |
| Plasma cells | 0.019 | * | 0.012 | * |
| B cells | 0.0048 | ** | 0.029 | * |
| T regulatory cells | 0.01 | ** | 0.026 | * |
| T cells CD4 | 0.033 | * | 0.086 | ns |
| Dendritic cells | 0.035 | * | 0.11 | ns |
| T cells CD8 | 0.035 | * | 0.12 | ns |
| Cytotoxic cells | 0.024 | * | 0.14 | ns |
| T cells gamma delta | 0.048 | * | 0.13 | ns |
| Macrophages M1 | 0.077 | ns | 0.24 | ns |
| NK cells | 0.13 | ns | 0.35 | ns |
| Mast cells | 0.51 | ns | 0.6 | ns |

| **Supplementary Table 3: PN gene expression changes between groups**  **Genes with lower expression in Group A than Group B in both metastatic and primary** | | | | | | | |
| --- | --- | --- | --- | --- | --- | --- | --- |
| AHSA2P | MKKS |  |  |  |  |  |  |
| ANKRD13C | OTUD3 |  |  |  |  |  |  |
| ATG12 | OTUD4 |  |  |  |  |  |  |
| ATG14 | OTUD6B |  |  |  |  |  |  |
| ATG2B | PFDN4 |  |  |  |  |  |  |
| ATG3 | PIK3C3 |  |  |  |  |  |  |
| ATG4C | PIK3CA |  |  |  |  |  |  |
| ATG5 | PPIG |  |  |  |  |  |  |
| ATXN3 | PPIL3 |  |  |  |  |  |  |
| BAG2 | PPIL4 |  |  |  |  |  |  |
| BAG4 | PPWD1 |  |  |  |  |  |  |
| BBS10 | PSMA2 |  |  |  |  |  |  |
| BBS12 | PSMA3 |  |  |  |  |  |  |
| C1GALT1C1 | PSMA4 |  |  |  |  |  |  |
| C1GALT1C1L | PSMA6 |  |  |  |  |  |  |
| CCT6B | PSMC6 |  |  |  |  |  |  |
| CDC37L1 | PSMD14 |  |  |  |  |  |  |
| CHORDC1 | PSME4 |  |  |  |  |  |  |
| CLPX | RNFT1 |  |  |  |  |  |  |
| DNAJA2 | SACS |  |  |  |  |  |  |
| DNAJB14 | SEL1L |  |  |  |  |  |  |
| DNAJB4 | SELENOF |  |  |  |  |  |  |
| DNAJB9 | SGTB |  |  |  |  |  |  |
| DNAJC1 | SPATA5 |  |  |  |  |  |  |
| DNAJC10 | SUGT1 |  |  |  |  |  |  |
| DNAJC2 | TMEM126B |  |  |  |  |  |  |
| DNAJC21 | TMX3 |  |  |  |  |  |  |
| DNAJC24 | TPP2 |  |  |  |  |  |  |
| DNAJC25 | UBE2B |  |  |  |  |  |  |
| DNAJC27 | UBE2D1 |  |  |  |  |  |  |
| DNAJC28 | UBE2J1 |  |  |  |  |  |  |
| DNAJC3 | UBE2V2 |  |  |  |  |  |  |
| DNAJC7 | UBE2W |  |  |  |  |  |  |
| FKBP14 | UBE3A |  |  |  |  |  |  |
| FKBP5 | UBR1 |  |  |  |  |  |  |
| FNIP2 | UBR2 |  |  |  |  |  |  |
| GRPEL2 | UBR3 |  |  |  |  |  |  |
| HSP90AA1 | UCHL5 |  |  |  |  |  |  |
| HSP90AA2P | USP14 |  |  |  |  |  |  |
| HSP90AA4P | USP25 |  |  |  |  |  |  |
| HSP90B1 | USP9X |  |  |  |  |  |  |
| HSP90B2P | VBP1 |  |  |  |  |  |  |
| HSPA13 | WDFY3 |  |  |  |  |  |  |
| HSPA14 | YOD1 |  |  |  |  |  |  |
| HSPA4L |  |  |  |  |  |  |  |
| LTN1 |  |  |  |  |  |  |  |
| MINDY2 |  |  |  |  |  |  |  |
| MINDY3 |  |  |  |  |  |  |  |

| **Genes with lower expression in Group A than Group B in primary and no difference in metastatic** | | | | | | | |
| --- | --- | --- | --- | --- | --- | --- | --- |
| BBS4 |  |  |  |  |  |  |  |
| CANX |  |  |  |  |  |  |  |
| CCT2 |  |  |  |  |  |  |  |
| CCT4 |  |  |  |  |  |  |  |
| DNAJA1 |  |  |  |  |  |  |  |
| DNAJC13 |  |  |  |  |  |  |  |
| DNAJC6 |  |  |  |  |  |  |  |
| DNAJC9 |  |  |  |  |  |  |  |
| ECPAS |  |  |  |  |  |  |  |
| HSPA12A |  |  |  |  |  |  |  |
| HSPD1 |  |  |  |  |  |  |  |
| HSPH1 |  |  |  |  |  |  |  |
| LAMP2 |  |  |  |  |  |  |  |
| MARCHF6 |  |  |  |  |  |  |  |
| NPM1 |  |  |  |  |  |  |  |
| OTUD1 |  |  |  |  |  |  |  |
| PDCL |  |  |  |  |  |  |  |
| PDIA6 |  |  |  |  |  |  |  |
| PIK3CB |  |  |  |  |  |  |  |
| PPID |  |  |  |  |  |  |  |
| PSMD10 |  |  |  |  |  |  |  |
| PSMD12 |  |  |  |  |  |  |  |
| PSMD5 |  |  |  |  |  |  |  |
| PSMD7 |  |  |  |  |  |  |  |
| PTGES3 |  |  |  |  |  |  |  |
| RAD23B |  |  |  |  |  |  |  |
| RNF103 |  |  |  |  |  |  |  |
| STT3B |  |  |  |  |  |  |  |
| UBE2D3 |  |  |  |  |  |  |  |
| UBE2E1 |  |  |  |  |  |  |  |
| UBE2E3 |  |  |  |  |  |  |  |
| UBE2K |  |  |  |  |  |  |  |
| UBE2Q2 |  |  |  |  |  |  |  |
| UBE3C |  |  |  |  |  |  |  |
| UBE4A |  |  |  |  |  |  |  |
| UBQLN1 |  |  |  |  |  |  |  |
| UBQLN2 |  |  |  |  |  |  |  |
| UBR5 |  |  |  |  |  |  |  |
| VMP1 |  |  |  |  |  |  |  |
|  |  |  |  |  |  |  |  |
|  |  |  |  |  |  |  |  |
|  |  |  |  |  |  |  |  |
|  |  |  |  |  |  |  |  |
|  |  |  |  |  |  |  |  |
|  |  |  |  |  |  |  |  |
|  |  |  |  |  |  |  |  |
|  |  |  |  |  |  |  |  |
|  |  |  |  |  |  |  |  |

| **Genes with higher expression in Group A than Group B in both metastatic and primary** | | | | | | | |
| --- | --- | --- | --- | --- | --- | --- | --- |
| FKBP8 |  |  |  |  |  |  |  |
| HSPB1 |  |  |  |  |  |  |  |
| HSPBP1 |  |  |  |  |  |  |  |
| SGTA |  |  |  |  |  |  |  |
| UBA52 |  |  |  |  |  |  |  |

| **Genes with higher expression in Group A than Group B in metastatic and no difference in primary** | | | | | | | |
| --- | --- | --- | --- | --- | --- | --- | --- |
| ADRM1 |  |  |  |  |  |  |  |
| ATG2A |  |  |  |  |  |  |  |
| ATG4D |  |  |  |  |  |  |  |
| ATG9A |  |  |  |  |  |  |  |
| BAG6 |  |  |  |  |  |  |  |
| BCAP31 |  |  |  |  |  |  |  |
| CNPY3 |  |  |  |  |  |  |  |
| DNAJC30 |  |  |  |  |  |  |  |
| DNAJC4 |  |  |  |  |  |  |  |
| ERP29 |  |  |  |  |  |  |  |
| GAK |  |  |  |  |  |  |  |
| OTUB1 |  |  |  |  |  |  |  |
| P4HB |  |  |  |  |  |  |  |
| PSMD2 |  |  |  |  |  |  |  |
| PSMD8 |  |  |  |  |  |  |  |
| RAD23A |  |  |  |  |  |  |  |
| SIL1 |  |  |  |  |  |  |  |
| SQSTM1 |  |  |  |  |  |  |  |
| STIP1 |  |  |  |  |  |  |  |
| STUB1 |  |  |  |  |  |  |  |
| TBCD |  |  |  |  |  |  |  |
| TIMM13 |  |  |  |  |  |  |  |
| TRAF2 |  |  |  |  |  |  |  |
| TRAP1 |  |  |  |  |  |  |  |
| TRIM11 |  |  |  |  |  |  |  |
| UBE2L5 |  |  |  |  |  |  |  |
| UBE2M |  |  |  |  |  |  |  |
| UBE2O |  |  |  |  |  |  |  |
| UBE2S |  |  |  |  |  |  |  |

| **Genes with lower expression in Group A than Group B in metastatic and no difference in primary** | | | | | | | |
| --- | --- | --- | --- | --- | --- | --- | --- |
| ATG16L2 |  |  |  |  |  |  |  |
| ATG4A |  |  |  |  |  |  |  |
| DNAAF4 |  |  |  |  |  |  |  |
| DNAJB7 |  |  |  |  |  |  |  |
| DNAJC19 |  |  |  |  |  |  |  |
| HSPA7 |  |  |  |  |  |  |  |
| LRP2BP |  |  |  |  |  |  |  |
| PFDN5 |  |  |  |  |  |  |  |
| PRKN |  |  |  |  |  |  |  |
| PSMA8 |  |  |  |  |  |  |  |
| UBE3D |  |  |  |  |  |  |  |

| **Genes with no difference in expression between Group A and B in primary or metastatic** | | | | | | | |
| --- | --- | --- | --- | --- | --- | --- | --- |
| AHSA1 | DNAJC14 | HSPA6 | PPIAL4F | RNF5 | UNC45B |  |  |
| AHSP | DNAJC15 | HSPA8 | PPIAL4G | SYVN1 | USP19 |  |  |
| AIPL1 | DNAJC16 | HSPA9 | PPIAL4H | TBCA | USP26 |  |  |
| AMFR | DNAJC17 | HSPB2 | PPIB | TBCC | USP4 |  |  |
| ATG10 | DNAJC18 | HSPB3 | PPIC | TCP1 | WDR45 |  |  |
| ATG101 | DNAJC22 | HSPB6 | PPIE | TIMM10 | WDR45B |  |  |
| ATG13 | DNAJC5 | HSPB7 | PPIF | TIMM44 | ZSWIM2 |  |  |
| ATG16L1 | DNAJC5B | HSPB8 | PPIH | TIMM8A |  |  |  |
| ATG4B | DNAJC5G | HSPB9 | PPIL1 | TIMM8B |  |  |  |
| ATG7 | DNAJC8 | HSPE1 | PSMA1 | TIMM9 |  |  |  |
| ATG9B | DNLZ | HUWE1 | PSMA5 | TOMM34 |  |  |  |
| ATXN3L | ERP27 | HYOU1 | PSMA7 | TOR1A |  |  |  |
| BAG1 | ERP44 | LRPAP1 | PSMB1 | TOR1B |  |  |  |
| BAG3 | FBXO2 | LRR1 | PSMB10 | TRAF3 |  |  |  |
| BAG5 | FBXO6 | LRRC29 | PSMB11 | TRIM13 |  |  |  |
| BCS1L | FKBP10 | LRRC41 | PSMB2 | TSACC |  |  |  |
| BECN1 | FKBP11 | MAP1LC3A | PSMB3 | TSC1 |  |  |  |
| BECN2 | FKBP15 | MAP1LC3B | PSMB4 | TTC4 |  |  |  |
| CALR | FKBP1A | MAP1LC3B2 | PSMB5 | UBA1 |  |  |  |
| CCT3 | FKBP1B | MAP1LC3C | PSMB6 | UBE2A |  |  |  |
| CCT5 | FKBP1C | MINDY1 | PSMB7 | UBE2C |  |  |  |
| CCT6A | FKBP2 | MINDY4 | PSMB8 | UBE2D2 |  |  |  |
| CCT7 | FK BP3 | NAA10 | PSMB9 | UBE2D4 |  |  |  |
| CCT8 | FKBP4 | NFE2L1 | PSMC1 | UBE2E2 |  |  |  |
| CCT8L1P | FKBP6 | OTUB2 | PSMC2 | UBE2F |  |  |  |
| CCT8L2 | FKBP7 | OTUD5 | PSMC3 | UBE2G1 |  |  |  |
| CDC34 | FKBP9 | OTUD6A | PSMC4 | UBE2G2 |  |  |  |
| CDC37 | FKBP9P1 | OTUD7A | PSMC5 | UBE2H |  |  |  |
| CHCHD4 | FKBPL | PAAF1 | PSMD1 | UBE2J2 |  |  |  |
| CLGN | GABARAP | PDCL3 | PSMD11 | UBE2L3 |  |  |  |
| CLNS1A | GABARAPL1 | PDIA2 | PSMD13 | UBE2L6 |  |  |  |
| CLU | GABARAPL2 | PDIA3 | PSMD3 | UBE2N |  |  |  |
| CRYAA | GET4 | PDIA4 | PSMD4 | UBE2NL |  |  |  |
| CRYAB | GRPEL1 | PDIA5 | PSMD6 | UBE2Q1 |  |  |  |
| CYBC1 | HDAC6 | PDRG1 | PSMD9 | UBE2QL1 |  |  |  |
| DNAJA3 | HOPX | PEX19 | PSME1 | UBE2R2 |  |  |  |
| DNAJA4 | HSCB | PFDN1 | PSME2 | UBE2T |  |  |  |
| DNAJB1 | HSP90AA5P | PFDN2 | PSME3 | UBE2U |  |  |  |
| DNAJB11 | HSP90AB1 | PFDN6 | PSMF1 | UBE2V1 |  |  |  |
| DNAJB12 | HSP90AB2P | PIK3C2B | PSMG1 | UBE2Z |  |  |  |
| DNAJB13 | HSP90AB3P | PIK3R2 | PSMG2 | UBE3B |  |  |  |
| DNAJB2 | HSPA12B | PIK3R4 | PSMG3 | UBE4B |  |  |  |
| DNAJB3 | HSPA1A | POMP | PSMG4 | UBQLN4 |  |  |  |
| DNAJB5 | HSPA1B | PPIA | RBX1 | UBR4 |  |  |  |
| DNAJB6 | HSPA1L | PPIAL4A | RCN3 | UBR7 |  |  |  |
| DNAJB8 | HSPA2 | PPIAL4C | RNF126 | UCHL1 |  |  |  |
| DNAJC11 | HSPA4 | PPIAL4D | RNF185 | UCHL3 |  |  |  |
| DNAJC12 | HSPA5 | PPIAL4E | RPS27A | ULK1 |  |  |  |

**Supplementary Table 4: Numbers of genes that are differentially expressed in CM that are differentially expressed in other cancers**

| **Study Abbreviation** | **Study Name** | **Number**  **of**  **Samples** | **Number of CM lower expressed genes with lower expression in one sample group than the other** | **Number of CM higher expressed genes with higher expression in one sample group than the other** | **Percentage of CM differentially expressed PN genes differentially expressed between groups** |
| --- | --- | --- | --- | --- | --- |
| CM | Skin Cutaneous Melanoma | 103 | 130 | 5 | 100.0 |
| UVM | Uveal Melanoma | 80 | 130 | 5 | 100.0 |
| UCEC | Uterine Corpus Endometrial Carcinoma | 587 | 127 | 4 | 97.0 |
| COAD | Colon adenocarcinoma | 521 | 120 | 5 | 92.6 |
| READ | Rectum adenocarcinoma | 177 | 115 | 4 | 88.1 |
| CESC | Cervical squamous cell carcinoma and endocervical adenocarcinoma | 309 | 106 | 3 | 80.7 |
| OV | Ovarian serous cystadenocarcinoma | 379 | 105 | 3 | 80.0 |
| LIHC | Liver hepatocellular carcinoma | 424 | 104 | 1 | 77.8 |
| THYM | Thymoma | 121 | 100 | 5 | 77.8 |
| BLCA | Bladder urothelial carcinoma | 433 | 99 | 4 | 76.3 |
| KICH | Kidney chromophobe | 89 | 99 | 2 | 74.8 |
| KIRP | Kidney renal papillary cell carcinoma | 321 | 97 | 4 | 74.8 |
| HNSC | Head and neck squamous cell carcinoma | 546 | 93 | 5 | 72.6 |
| KIRC | Kidney renal clear cell carcinoma | 611 | 91 | 4 | 70.4 |
| PRAD | Prostate adenocarcinoma | 551 | 88 | 5 | 68.9 |
| THCA | Thyroid carcinoma | 568 | 86 | 5 | 67.4 |
| LUAD | Lung adenocarcinoma | 594 | 81 | 5 | 63.7 |
| DLBC | Lymphoid Neoplasm Diffuse Large B-cell Lymphoma | 48 | 79 | 5 | 62.2 |
| BRCA | Breast invasive carcinoma | 1102 | 77 | 5 | 60.7 |
| PCPG | Pheochromocytoma and Paraganglioma | 186 | 72 | 3 | 55.6 |
| LGG | Brain Lower Grade Glioma | 529 | 67 | 4 | 52.6 |
| LAML | Acute Myeloid Leukemia | 151 | 66 | 1 | 49.6 |
| MESO | Mesothelioma | 86 | 66 | 1 | 49.6 |
| STAD | Stomach adenocarcinoma | 407 | 63 | 3 | 48.9 |
| TGCT | Testicular Germ Cell Tumors | 156 | 63 | 2 | 48.1 |
| SARC | Sarcoma | 265 | 58 | 2 | 44.4 |
| ESCA | Oesophageal carcinoma | 173 | 44 | 2 | 34.1 |
| LUSC | Lung squamous cell carcinoma | 551 | 43 | 0 | 31.9 |
| GBM | Glioblastoma multiforme | 174 | 36 | 0 | 26.7 |
| UCS | Uterine Carcinosarcoma | 56 | 34 | 1 | 25.9 |
| CHOL | Cholangiocarcinoma | 46 | 18 | 0 | 13.3 |

**Supplementary Table 5: classes of regulatory function executed by key regulators**

| Regulators​ | DNA  Binding​ | DNA  Methylation​ | RNA  Binding​ | Protein  Binding​ | Histone Acetylation  and Methylation​ | Chromatin Remodelling  And Organisation​ | DNA Damage Response  and  Repair​ |
| --- | --- | --- | --- | --- | --- | --- | --- |
| RB1CC1​ | ​ | ​ | ​ | Yes​ | ​ | ​ | ​ |
| ARID4A​ | Yes​ | Yes​ | Yes​ | ​ | Yes​ | Yes​ | ​ |
| ZRANB2​ | Yes​ | ​ | Yes​ | ​ | Yes​ | ​ | ​ |
| ARID4B​ | Yes​ | Yes​ | Yes​ | ​ | Yes​ | Yes​ | ​ |
| ZMYND11​ | Yes​ | Yes​ | ​ | Yes​ | ​ | Yes​ | ​ |
| HLTF​ | Yes​ | Yes​ | Yes​ | Yes​ | ​ | Yes​ | Yes​ |
| RBM7​ | ​ | Yes​ | Yes​ | Yes​ | Yes​ | ​ | ​ |
| PMS1​ | Yes​ | ​ | ​ | Yes​ | ​ | ​ | Yes​ |
| SIRT1​ | Yes​ | Yes​ | ​ | Yes​ | Yes​ | Yes​ | Yes​ |
| TMF1​ | Yes​ | ​ | ​ | ​ | ​ | ​ | ​ |
| GCFC2​ | Yes​ | ​ | ​ | ​ | ​ | ​ | ​ |
| YAF2​ | Yes​ | ​ | ​ | ​ | Yes​ | ​ | ​ |
| ZNF451​ | ​ | Yes​ | ​ | ​ | Yes​ | Yes​ | ​ |
| NR3C1​ | Yes​ | Yes​ | Yes​ | Yes​ | Yes​ | Yes​ | ​ |
| RNF19A​ | ​ | ​ | ​ | Yes​ | ​ | ​ | ​ |
| SP4​ | Yes​ | ​ | ​ | ​ | ​ | ​ | ​ |
| DDX5​ | Yes​ | ​ | Yes​ | Yes​ | Yes​ | ​ | Yes​ |
| ZBTB11​ | Yes​ | ​ | ​ | ​ | ​ | ​ | ​ |
| CREB1​ | Yes​ | ​ | ​ | Yes​ | Yes​ | ​ | Yes​ |
| SP3​ | Yes​ | ​ | ​ | ​ | Yes​ | ​ | ​ |
| SNAPC1​ | Yes​ | ​ | ​ | ​ | ​ | ​ | ​ |
| ZNF148​ | Yes​ | ​ | ​ | ​ | Yes​ | ​ | ​ |
| ELK3​ | Yes​ | ​ | ​ | ​ | ​ | ​ | ​ |
| GABPA​ | Yes​ | ​ | ​ | Yes​ | ​ | ​ | ​ |
| CEBPZ​ | Yes​ | ​ | Yes​ | ​ | Yes​ | ​ | ​ |
| TRIP11​ | ​ | ​ | ​ | ​ | Yes​ | ​ | ​ |
| ATF2​ | Yes​ | ​ | ​ | Yes​ | Yes​ | Yes​ | Yes​ |
| MED13​ | Yes​ | ​ | ​ | Yes​ | ​ | ​ | ​ |
| FMR1​ | ​ | Yes​ | Yes​ | Yes​ | Yes​ | Yes​ | Yes​ |
| DMTF1​ | Yes​ | ​ | ​ | ​ | ​ | ​ | ​ |
| MEF2A​ | Yes​ | ​ | ​ | Yes​ | Yes​ | ​ | ​ |
| SKIL​ | Yes​ | ​ | ​ | Yes​ | ​ | ​ | Yes​ |
| BTAF1​ | Yes​ | ​ | ​ | ​ | Yes​ | ​ | ​ |
| ZNF638​ | Yes​ | Yes​ | Yes​ | ​ | ​ | Yes​ | ​ |
| ATRX​ | Yes​ | Yes​ | ​ | Yes​ | Yes​ | Yes​ | Yes​ |
| DEK​ | Yes​ | ​ | Yes​ | Yes​ | Yes​ | Yes​ | Yes​ |
| CRY1​ | Yes​ | ​ | ​ | Yes​ | Yes​ | ​ | Yes​ |
| ERCC8​ | ​ | ​ | ​ | Yes​ | ​ | ​ | Yes​ |
| NAB1​ | ​ | ​ | ​ | Yes​ | ​ | ​ | ​ |
| PNN​ | Yes​ | Yes​ | Yes​ | ​ | Yes​ | ​ | ​ |
| SMARCE1​ | Yes​ | Yes​ | Yes​ | Yes​ | Yes​ | Yes​ | ​ |
| ZMYM2​ | Yes​ | ​ | ​ | Yes​ | ​ | ​ | ​ |
| CNOT3​ | ​ | ​ | ​ | ​ | ​ | ​ | Yes​ |
| ZNF510​ | Yes​ | ​ | ​ | ​ | ​ | ​ | ​ |
| ZFX​ | Yes​ | ​ | ​ | ​ | ​ | ​ | ​ |
| MATR3​ | ​ | ​ | Yes​ | Yes​ | Yes​ | ​ | ​ |
| RLF​ | Yes​ | Yes​ | ​ | ​ | Yes​ | Yes​ | ​ |
| NFYB​ | Yes​ | ​ | ​ | Yes​ | ​ | ​ | ​ |
| MLLT10​ | Yes​ | ​ | Yes​ | Yes​ | ​ | ​ | ​ |

| **DNA Binding** |
| --- |
| ARID DNA-binding domain |
| ARID DNA-binding domain superfamily |
| Cis-regulatory region sequence-specific DNA binding |
| Core promoter sequence-specific DNA binding |
| DNA binding |
| DNA-binding |
| DNA-binding transcription activator activity |
| DNA-binding transcription activator activity, RNA polymerase II-specific |
| DNA-binding transcription factor activity |
| DNA-binding transcription factor activity, RNA polymerase II-specific |
| DNA-binding transcription factor binding |
| DNA-binding transcription repressor activity |
| DNA-binding transcription repressor activity, RNA polymerase II-specific |
| Double-stranded DNA binding |
| Lambda repressor-like, DNA-binding domain superfamily |
| Myb-like DNA-binding domain |
| p53-like transcription factor, DNA-binding |
| Positive regulation of transcription regulatory region DNA binding |
| Regulation of transcription regulatory region DNA binding |
| RNA polymerase II cis-regulatory region sequence-specific DNA binding |
| RNA polymerase II core promoter sequence-specific DNA binding |
| RNA polymerase II transcription regulatory region sequence-specific DNA binding |
| RNA polymerase II-specific DNA-binding transcription factor binding |
| SANT SWI3, ADA2, N-CoR and TFIIIB DNA-binding domains |
| Sequence-specific DNA binding |
| Sequence-specific double-stranded DNA binding |
| Single-stranded DNA binding |
| Transcription regulatory region sequence-specific DNA binding |

| **DNA Methylation** |
| --- |
| DNA methylation |
| DNA methylation or demethylation |
| methyl-CpG binding |
| Methyl-CpG binding domain |
| Methylation |
| Methyltransferase |
| Methyltransferase activity |
| Methyltransferase complex |

**Supplementary Table 6: Allocation of GO terms to functional categories**

| **DNA Damage Response and Repair** |
| --- |
| Cellular response to DNA damage stimulus |
| Damaged DNA binding |
| DNA damage |
| DNA damage checkpoint |
| DNA Damage Recognition in GG-NER |
| DNA damage response |
| DNA damage response (only ATM dependent) |
| DNA damage response, signal transduction by p53 class mediator |
| DNA damage response, signal transduction by p53 class mediator resulting in cell cycle arrest |
| DNA damage response, signal transduction by p53 class mediator resulting in transcription of p21 class mediator |
| DNA damage response, signal transduction resulting in transcription |
| DNA Damage/Telomere Stress Induced Senescence |
| DNA Double Strand Break Response |
| DNA double-strand break processing |
| DNA Double-Strand Break Repair |
| DNA integrity checkpoint |
| DNA IR-damage and cellular response via ATR |
| DNA IR-double strand breaks and cellular response via ATM |
| DNA repair |
| DNA Repair |
| DNA repair complex |
| DNA repair complex, and DNA N-glycosylase activity |
| DNA repair pathways, full network |
| DNA replication, and interstrand cross-link repair |
| Double-strand break repair |
| Double-strand break repair via break-induced replication |
| Double-strand break repair via nonhomologous end joining |
| G1 DNA damage checkpoint |
| G2 DNA damage checkpoint |
| G2/M DNA damage checkpoint |
| Gap-filling DNA repair synthesis and ligation in TC-NER |
| Homologous DNA Pairing and Strand Exchange |
| Intracellular signal transduction involved in G1 DNA damage checkpoint |
| Intrinsic apoptotic signaling pathway in response to DNA damage |
| Intrinsic apoptotic signaling pathway in response to DNA damage by p53 class mediator |
| miRNA regulation of DNA damage response |
| Mitotic DNA damage checkpoint |
| Mitotic G1 DNA damage checkpoint |
| Nucleotide-excision repair, DNA duplex unwinding |
| Nucleotide-excision repair, DNA incision |
| Nucleotide-excision repair, DNA incision, 5-to lesion |
| Positive regulation of DNA repair |
| Positive regulation of response to DNA damage stimulus |
| Processing of DNA double-strand break ends |
| Regulation of DNA repair |
| Regulation of intrinsic apoptotic signaling pathway in response to DNA damage |
| Regulation of response to DNA damage stimulus |
| Signal transduction in response to DNA damage |
| Signal transduction involved in DNA damage checkpoint |
| Signal transduction involved in mitotic DNA damage checkpoint |
| Signal transduction involved in mitotic DNA integrity checkpoint |
| Signal transduction involved in mitotic G1 DNA damage checkpoint |
| SUMOylation of DNA damage response and repair proteins |
| TP53 Regulates Transcription of DNA Repair Genes |

| **Histone acetylation and methylation** |
| --- |
| Acetylation |
| Acetylation-dependent protein binding |
| Acetylation-dependent protein binding |
| Acetyltransferase activity |
| Acetyltransferase complex |
| H3 histone acetyltransferase complex |
| H3 histone acetyltransferase complex, and histone H4-K12 acetylation |
| H4 histone acetyltransferase complex |
| H4 histone acetyltransferase complex, and INO80-type complex |
| H4 histone acetyltransferase complex, and Swr1 complex |
| H4/H2A histone acetyltransferase complex |
| HDACs deacetylate histones |
| HDACs deacetylate histones, and Methyl-CpG binding domain |
| HDACs deacetylate histones, and NoRC negatively regulates rRNA expression |
| HDACs deacetylate histones, and Sin3-type complex |
| Histone acetylation |
| Histone acetyltransferase activity |
| Histone acetyltransferase binding |
| Histone acetyltransferase complex |
| Histone deacetylase binding |
| Histone deacetylase complex |
| Histone deacetylation |
| Histone demethylase activity |
| Histone demethylase activity (H3-K9 specific), and histone methyltransferase activity (H4-K20 specific) |
| Histone demethylation |
| Histone H3 acetylation |
| Histone H3 deacetylation |
| Histone H3-K27 methylation |
| Histone H3-K4 methylation |
| Histone H3-K9 methylation |
| Histone H4 acetylation |
| Histone H4-K12 acetylation |
| Histone H4-K20 methylation |
| Histone lysine demethylation |
| Histone lysine methylation |
| Histone lysine methylation, and JmjC domain, hydroxylase |
| Regulation of histone H4 acetylation |
| Regulation of histone methylation |
| Regulation of peptidyl-lysine acetylation |
| Regulation of peptidyl-lysine acetylation |
| Regulation of protein acetylation |
| Regulation of protein acetylation |
| Regulation of protein deacetylation |
| Regulation of protein deacetylation |
| Regulation of TP53 Activity through Acetylation |
| RMTs methylate histone arginines |
| S-adenosylmethionine-dependent methyltransferase activity |

| **Chromatin Remodelling and Organisation** |
| --- |
| ATP-dependent chromatin remodeling |
| CENP-A containing chromatin organization |
| Chromatin assembly |
| Chromatin assembly or disassembly |
| Chromatin disassembly |
| Chromatin modifying enzymes |
| Chromatin organization |
| Chromatin organization involved in negative regulation of transcription |
| Chromatin organization involved in regulation of transcription |
| Chromatin organization modifier domain |
| Chromatin regulator |
| Chromatin remodeling |
| Chromatin remodeling at centromere |
| Chromatin silencing |
| Chromatin-mediated maintenance of transcription |
| Chromatin-mediated maintenance of transcription |
| Covalent chromatin modification |
| Covalent chromatin modification |
| DNA methylation-dependent heterochromatin assembly |
| Heterochromatin assembly |
| Heterochromatin organization |
| Negative regulation of chromatin organization |
| Positive regulation of chromatin assembly or disassembly |
| Positive regulation of chromatin organization |
| Positive regulation of DNA methylation-dependent heterochromatin assembly |
| Positive regulation of heterochromatin assembly |
| rDNA heterochromatin assembly |
| Regulation of chromatin assembly or disassembly |
| Regulation of chromatin organization |
| Regulation of DNA methylation-dependent heterochromatin assembly |
| Regulation of heterochromatin assembly |
| SUMOylation of chromatin organization proteins |
| Regulation of histone H3-K4 methylation |
| Regulation of histone H3-K9 methylation   \| **Protein Binding** \| \| --- \| \| Cytoskeletal protein binding \| \| Enzyme binding \| \| Heat shock protein binding \| \| Identical protein binding \| \| Kinase binding \| \| Protein binding \| \| Protein C-terminus binding \| \| Protein kinase binding \| \| Protein N-terminus binding \| \| Protein phosphatase binding \| \| Protein-containing complex binding \| \| Ubiquitin protein ligase binding \| |

| **RNA Binding** |
| --- |
| miRNA binding |
| Primary miRNA binding |
| Regulatory RNA binding |
| RNA binding |
| Single-stranded RNA binding |
